# Quantitative N-glycoproteomics reveals altered glycosylation levels of various plasma proteins in bloodstream infected patients

**DOI:** 10.1371/journal.pone.0195006

**Published:** 2018-03-29

**Authors:** Sakari Joenvaara, Mayank Saraswat, Pentti Kuusela, Shruti Saraswat, Rahul Agarwal, Johanna Kaartinen, Asko Järvinen, Risto Renkonen

**Affiliations:** 1 Transplantation laboratory, Haartmaninkatu 3, University of Helsinki, Helsinki, Finland; 2 HUSLAB, Helsinki University Hospital, Helsinki, Finland; 3 Division of Clinical Microbiology, HUSLAB, Helsinki, Finland; 4 Department of Bacteriology and Immunology, University of Helsinki, Helsinki, Finland; 5 Department of Reproductive Biology, All India Institute of Medical Sciences, New Delhi, India; 6 Emergency Medicine and Services, Helsinki University Hospital, Helsinki, Finland; 7 Division of Infectious Diseases, HUH Inflammation Center, University of Helsinki, Helsinki, Finland; NIH, UNITED STATES

## Abstract

Bloodstream infections are associated with high morbidity and mortality with rates varying from 10–25% and higher. Appropriate and timely onset of antibiotic therapy influences the prognosis of these patients. It requires the diagnostic accuracy which is not afforded by current gold standards such as blood culture. Moreover, the time from blood sampling to blood culture results is a key determinant of reducing mortality. No established biomarkers exist which can differentiate bloodstream infections from other systemic inflammatory conditions. This calls for studies on biomarkers potential of molecular profiling of plasma as it is affected most by the molecular changes accompanying bloodstream infections. N-glycosylation is a post-translational modification which is very sensitive to changes in physiology. Here we have performed targeted quantitative N-glycoproteomics from plasma samples of patients with confirmed positive blood culture together with age and sex matched febrile controls with negative blood culture reports. Three hundred and sixty eight potential N-glycopeptides were quantified by mass spectrometry and 149 were further selected for identification. Twenty four N-glycopeptides were identified with high confidence together with elucidation of the peptide sequence, N-glycosylation site, glycan composition and proposed glycan structures. Principal component analysis, orthogonal projections to latent structures-discriminant analysis (S-Plot) and self-organizing maps clustering among other statistical methods were employed to analyze the data. These methods gave us clear separation of the two patient classes. We propose high-confidence N-glycopeptides which have the power to separate the bloodstream infections from blood culture negative febrile patients and shed light on host response during bacteremia. Data are available via ProteomeXchange with identifier PXD009048.

## Introduction

Bloodstream infections are an increasing problem which is correlated to high morbidity and mortality[1]. Bacteremic infections even today are associated with high mortality which depending on the causative agent varies between 10–25% but in most severe cases may be significantly higher [1, 2]. Early diagnosis and onset of effective antibiotic therapy is the key in reducing the mortality in bloodstream bacterial infections[3]. However, antibiotic treatment in bacteremic infections has to be initiated empirically based on clinical findings viewed against unspecific laboratory markers of inflammation. Plasma levels of circulated inflammatory mediators such as C-reactive protein (CRP), procalcitonin (PCT) and interleukin-6 (IL-6) have been suggested to predict the bacterial bloodstream infections[4–6]. However, these biomarkers are not sufficiently sensitive or specific because they are elevated in other non-infectious including trauma and inflammation for example due to a rheumatological disease[7–9]. The golden standard in diagnosis of bacteremic infections is blood culture which is far too slow for early clinical decisions as the results have to be waited for 24 to 48 hours. This results in unnecessary antibiotic therapies with risk for resistance development. On the other hand, those patients with a true positive blood culture may not be treated efficiently enough. Novel markers specific for bloodstream infections might help to direct therapy to right patients from the very early onset of the disease when it would be most desperately needed.

Plasma proteomics as well as glycoproteomics has been suggested to be an important source of biomarkers for systemic diseases [10–12]. Our study on the subject was able to suggest multiple protein markers of bloodstream infection[12]. Post-translational modification (PTM) of proteins increase the repertoire of functions proteins can perform. One of the most abundant PTM, glycosylation is found in about 50% of human proteins and covalent addition of glycan residues to asparagine is called N-glycosylation. Changes in level or sites of N-glycosylation can alter the physiological function of proteins or lead to patho-physiological processes. N-glycosylation machinery is very sensitive to changes in physiology caused by pathological processes such as cancer or microbial infection[11, 13]. These changes can be detected and quantified to work as detection and monitoring biomarkers of pathological processes.

There are abundant changes in plasma proteins due to severe inflammatory reactions. Glycoproteomic analyses have been shown to discern survivors from non-survivors due to sepsis in ICU [11]. We have previously developed a quantitative N-glycoproteomics workflow in a series of studies[14–17]. This workflow can lead to quantification of N-glycopeptides and at the same time establish their peptide sequence, glycan composition and proposed glycan structure[14–17]. In the current study, extending our proteomic findings[12], we utilize this workflow to quantify the N-glycopeptides in samples from patients having a confirmed bloodstream infection and age and sex matched febrile non-bacteremic controls. The quantification was followed by stringent statistical analyses such as ANOVA, Principal component analysis (PCA) and Orthogonal projections to latent structures-discriminant analysis (OPLS-DA). OPLS-DA analysis gave us the S-Plot which was used to find the significantly differing proteins among the bloodstream infection individuals and controls in bacterial strain-independent manner. Three hundred sixty eight potential N-glycopeptide ions were quantified and 149 were selected to be fragmented and identified. We identified 24 N-glycopeptides along with their peptide sequence, N-glycosylation site, glycan composition and proposed glycan structures. As shown by various statistical analyses, these N-glycopeptide ions were able to classify bloodstream infected cases from febrile non-bacteremic controls.

## Results

### Overview

**Fig 1:**
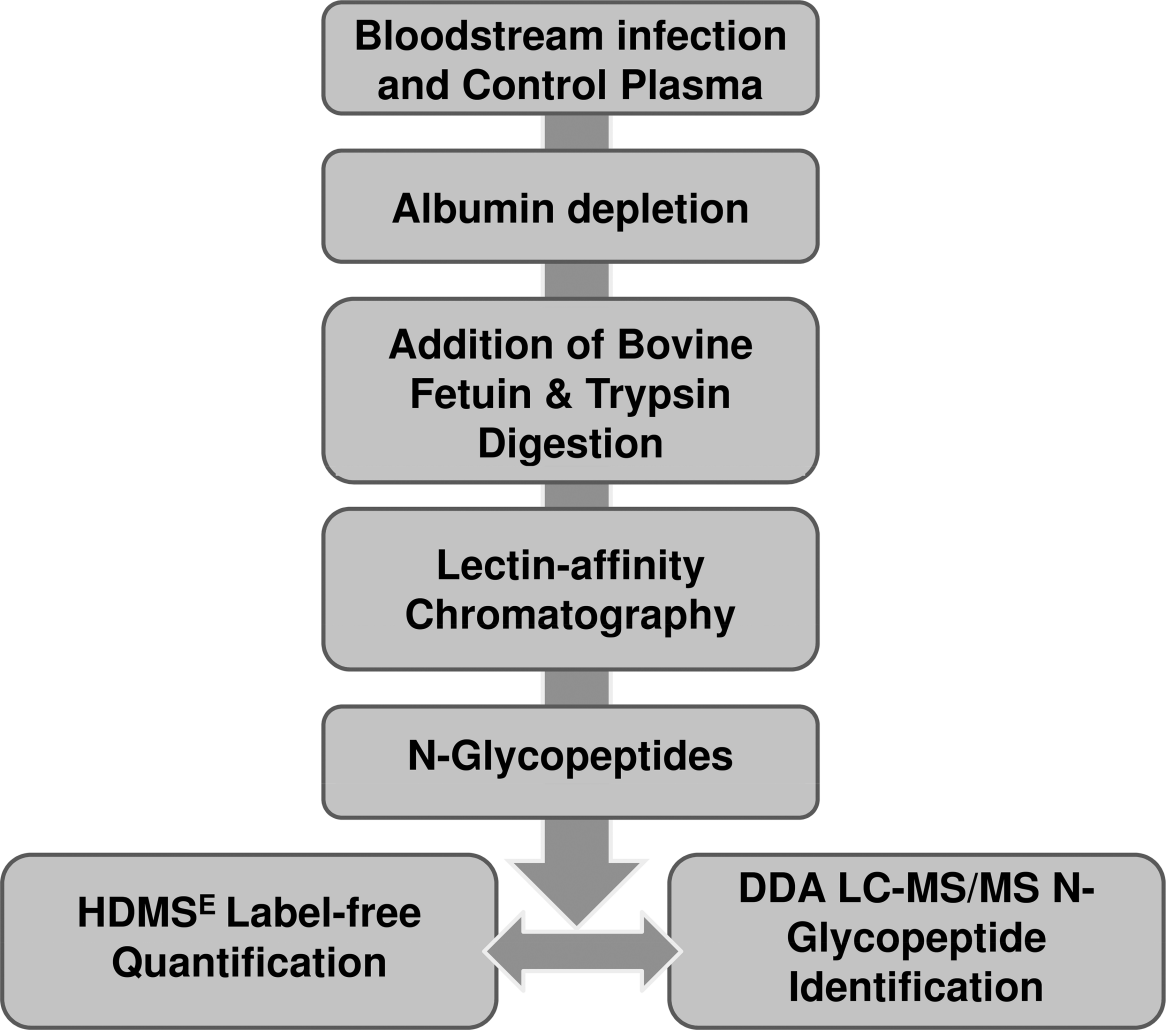
Overview of the workflow. The complete workflow starting form plasma samples to quantification and identification of N-glycopeptides is summarized here.

The whole workflow utilized interconnected steps in a sequential manner which is presented in Fig 1. Plasma samples from ten blood culture confirmed cases of bloodstream infection and ten age and sex matched febrile controls were obtained as described in methods. These samples were processed to enrich N-glycopeptides which were analyzed in two sequential but separate steps: Step 1: HDMS^E^ to quantify the precursor ions (m/z) and; Step 2: LC-MS/MS to identify the N-glycopeptides as well as to establish the peptide sequence, glycan composition and proposed glycan structure for those precursors which differed significantly between the cases and controls. Publicly available software package GlycopepitdeId was used for searching the LC-MS/MS spectra peak list for step 2.

### Patients

Twenty febrile patients coming to Peijas hospital (Vantaa, Finland) were divided into two groups based on blood culture findings (blood culture positive and blood culture negative). Blood culture positive group included 8 men and 2 women and was designated “cases” while 10 blood culture negative patients (age and sex matched) were designated as controls. Blood cultures identified bacterial species were *Escherichia coli* (4 samples), *Streptococcus pneumoniae* (2 samples), coagulase-negative Staphylococcus (2 samples), *Staphylococcus epidermidis* and *Streptococcus viridans* (1 sample each). Plasma C-reactive protein (CRP) levels among cases were 12–399 mg/L and febrile controls had levels of CRP ranging from 13–101 mg/L. Among the control patients, four patients presented with no verified infection and were not treated with antibiotics. Two patients had pyelonephritis and four patients presented with a respiratory infection which was supposed to be of viral origin. However, three of the controls supposedly having respiratory viral infection received oral antibiotics (doxycycline, cephalexin or co-amoxyclavulanate). All HDMS^E^ runs performed well in terms of chromatographic performance and alignment and therefore no sample was excluded from analysis.

### HDMS^E^ and statistical analysis

N-glycopeptides were enriched from tryptic digests of albumin-depleted total plasma proteins utilizing a mixture of four different lectins. Concanavalin A (Con A), Sambucus nigra agglutinin (SNA), Lens culinaris (LCA) and Aleuria aurantia lectin (AAL) slurries were mixed in a ratio of 5:3:3:1 and N-glycopeptides were enriched from tryptic digests mixture. This ratio of lectin was empirically determined (data not shown). The presence of N-glycopeptides was verified in the high energy function of MS^E^ runs by characteristic N-glycan fragmentation ions (m/z values of 138, 204, 366.14, 657.23 etc.). Every LC-MS run was performed in triplicate.

**Fig 2:**
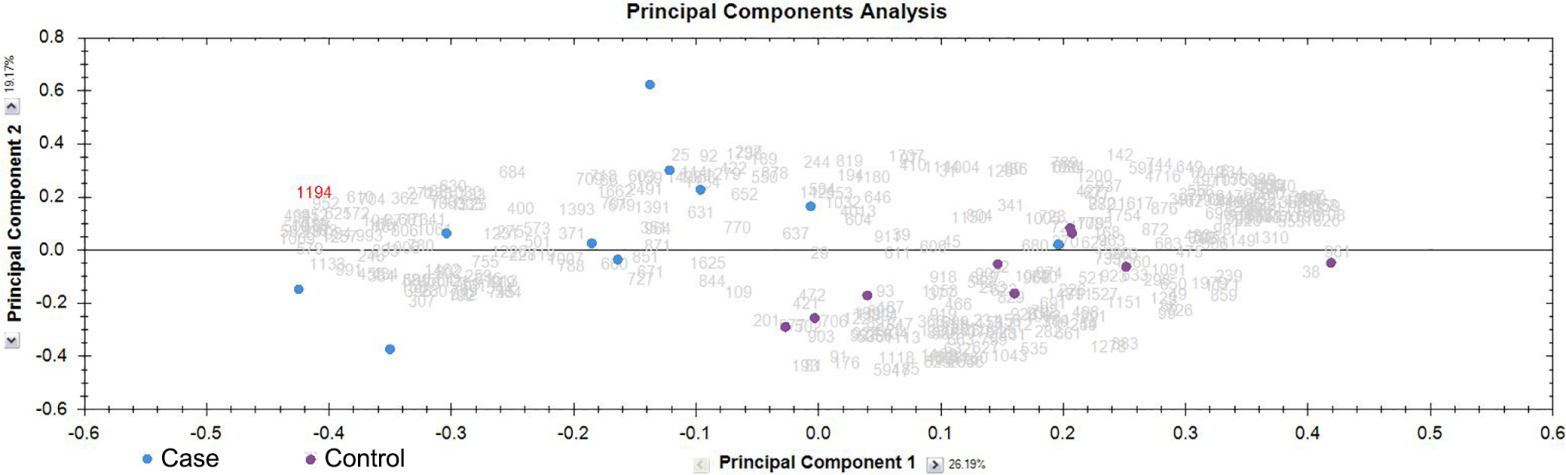
Principal component analysis. Principal component analysis of all the quantified potential N-glycopeptide ions was performed with the Progenesis software. Blue circles represent blood culture positive cases while the purple circles are febrile controls. X-axis is principal component 1 and Y-axis is principal component 2.

Raw data was imported into Progenesis and peak picking and alignment was performed as described in the methods. Potential glycopeptides were selected for further analysis with following criteria: requirement for charge of +3 to +5 and intensity of at least 10^4^. Three hundred sixty eight precursor ions matched these criteria and they were quantified (Table A in S2 File). T-test was performed on this data and the resulting p values are given in Table A in S2 File. This quantitative data was used for performing principal component analysis (PCA) which is shown in Fig 2.

**Fig 3:**
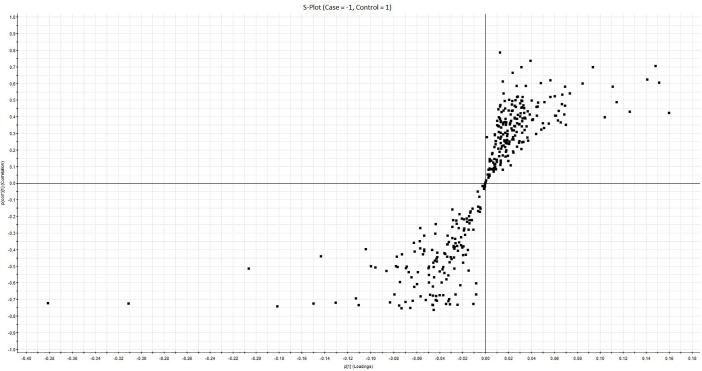
S-plot. Orthogonal projections to latent structures-discriminant analysis modeling was performed with the software EZInfo 3.0 and resulting S-plot is shown here. X-axis is loadings and Y-axis is correlation. Minus half of the figure is proteins having higher expression in cases while plus half is the proteins having higher expression in controls.

PCA based on 368 quantified ions gave separation of cases vs controls. However, one case was segregating from the other cases and clustering with the control class (Fig 2). To further identify the features (Ions) which can classify the cases vs controls, the data was used to perform the orthogonal projections to latent structures-discriminant analysis (OPLS-DA). This modeling of the data gave us the S-Plot which is shown in Fig 3.

OPLS-DA is a modeling technique which can identify the classifying features of two groups of samples representing predictive variance. These features (ions) can then be used to classify the sample groups into their respective classes (for example cases vs controls). Forty one ions were selected as being significantly different between the cases and controls (p(Corr) values given in Table B in S2 File). When we used S-plot ions for running PCA analysis, we were able to completely separate the cases and controls including the one case sample (Case 10) which was not separating that well with other analyses (Figure A in S1 File). These S-Plot ions correspond to a panel which can be used in targeted assays to predict the blood culture positive patients and separate them from other febrile patients however further studies are needed in this regard.

### Identification of N-glycopeptides

Based on S-Plot, T-test p values and intensity in HDMS^E^ runs, 149 ions were selected to be fragmented for identifying the peptide sequence, N-glycosylation site, N-glycan sequence and proposed N-glycan structure. Out of these 149 ions, we were able to confidently identify 24 glycopeptide ions with a false discovery rate (FDR) of 3.84%. These glycopeptides are presented in Table 1. The median percentage coefficient of variation for all triplicate run was 4.011% (for t-test p value <0.05). This suggests robust technical reproducibility of the runs. Cases had within-group median %CV at 65.54%, and controls 50.26% (t-test p value <0.05). This suggests that inter-individual variability of N-glycopeptides is high which is however, in accordance with previous reports [18].

**Table 1:** Identified N-glycopeptides. m/z values of the N-glycopeptides, cgharrge, Uniport Id, peptide mass, N-glycosylation site, peptide sequence, Glycan composition, porposed annotated structure, N-glycopeptide scores and area under the curve (AUC) values from Receiver operating curve analysis of these N-glycopeptides are given in the table. Where a structure was not matched with database and was elucidated de novo, it is written as denovo (see methods and references therein for more details).

m/z	Charge	Protein Uniprot Id	Peptide_Mass	N_site	Peptide Sequence	Glycan composition	Proposed Glycan Structure	Score	AUC from ROC curve analysis
990.9476	4	A1AT_HUMAN	1754.888	271	YLGNATAIFFLPDEGK	S2H5N4	SHNH(SHNH)HNN	77.59	0.860
1011.432	4	A1AT_HUMAN	1754.888	271	YLGNATAIFFLPDEGK	S2H3N6	denovo	44.72	0.857
1320.927	3	A1AT_HUMAN	1754.888	271	YLGNATAIFFLPDEGK	S2H5N4	SHNH(SHNH)HNN	70.66	0.884
1068.449	4	CERU_HUMAN	1891.834	138	EHEGAIYPDNTTDFQR	H5N7F1	denovo	40.55	0.774
1067.238	5	CGAT1_HUMAN	2316.176	324	AANFRNFTFIQLNGEFSRGK	S2H10N4	denovo	28.54	0.782
903.1394	4	HEMO_HUMAN	1403.674	187	SWPAVGNCSSALR	S2H5N4	SHNH(SHNH)HNN	51.02	0.568
939.653	4	HEMO_HUMAN	1403.674	187	SWPAVGNCSSALR	S2H5N4F1	SHNH(SHNH)HN(F)N	46.03	0.753
985.9345	4	HEMO_HUMAN	1734.885	453	ALPQPQNVTSLLGCTH	S2H5N4	SHNH(SHNH)HNN	39.93	0.787
1203.814	3	HEMO_HUMAN	1403.674	187	SWPAVGNCSSALR	S2H5N4	SHNH(SHNH)HNN	77.43	0.596
932.0305	5	HPT_HUMAN	1794.004	241	VVLHPNYSQVDIGLIK	S3H6N5	SHNH(SHN(SHN)H)HNN	39.03	0.887
1092.015	4	HPT_HUMAN	1794.004	241	VVLHPNYSQVDIGLIK	S2H6N5	SHNH(SHN(HN)H)HNN	39.04	0.764
1115.226	3	HPT_HUMAN	1794.004	241	VVLHPNYSQVDIGLIK	S1H4N3	SHNH(H)HNN	58.34	0.900
1225.552	4	HPT_HUMAN	1794.004	241	VVLHPNYSQVDIGLIK	S2H10N3F2	denovo	42.13	0.641
1333.97	3	HPT_HUMAN	1794.004	241	VVLHPNYSQVDIGLIK	S2H5N4	SHNH(SHNH)HNN	74.33	0.910
916.6563	4	HPTR_HUMAN	1457.726	149,153	NLFLNHSENATAK	S2H5N4	SHNH(SHNH)HNN	64.48	0.868
885.6227	5	IGHA1_HUMAN	2962.604	144	LSLHRPALEDLLLGSEANLTCTLTGLR	H4N4	HNH(NH)HNN	29.79	0.767
976.2765	5	IGHA1_HUMAN	2962.604	144	LSLHRPALEDLLLGSEANLTCTLTGLR	S1H5N4	SHNH(HNH)HNN	36.56	0.618
984.488	5	IGHA1_HUMAN	2962.604	144	LSLHRPALEDLLLGSEANLTCTLTGLR	S1H4N5	SHNH(NH)(N)HNN	54.39	0.789
1016.904	5	IGHA1_HUMAN	2962.604	144	LSLHRPALEDLLLGSEANLTCTLTGLR	S1H5N5	SHN(HN)H(NH)HNN	64.97	0.954
1147.326	4	IGHA1_HUMAN	2962.604	144	LSLHRPALEDLLLGSEANLTCTLTGLR	H5N4	HNH(HNH)HNN	33.31	0.723
1179.592	4	IGHA1_HUMAN	2962.604	144	LSLHRPALEDLLLGSEANLTCTLTGLR	S1H4N4	SHNH(NH)HNN	60.04	0.713
868.048	3	IGHG2_HUMAN	1156.515	176	EEQFNSTFR	H3N4F1	NH(NH)HN(F)N	85.11	0.767
885.1539	4	IGHG2_HUMAN	1638.811	176	TKPREEQFNSTFR	S1H4N4F1	SHNH(NH)HN(F)N	42.52	0.856
989.7621	3	IGHG2_HUMAN	1156.515	176	EEQFNSTFR	H4N5F1	NNH(HNH)HN(F)N	62.53	0.623

These 24 glycopeptides belonged to 8 different proteins. Ten unique peptides were found containing 11 N-glycosylation consensus site (NXS/T/C, where X is any amino acid except proline). One peptide contained two consensus sites and the occupancy of either site was ambiguous. Peptide identification scores ranged from 21.75 to 50.18. Data-dependent acquisition LC-MS was performed in a targeted way to identify the glycopeptide ions. We have employed this N-glycopeptide identification workflow in our previous studies in different settings and set of samples[15–17].

### Glycan compositions and proposed structures

**Fig 4:**
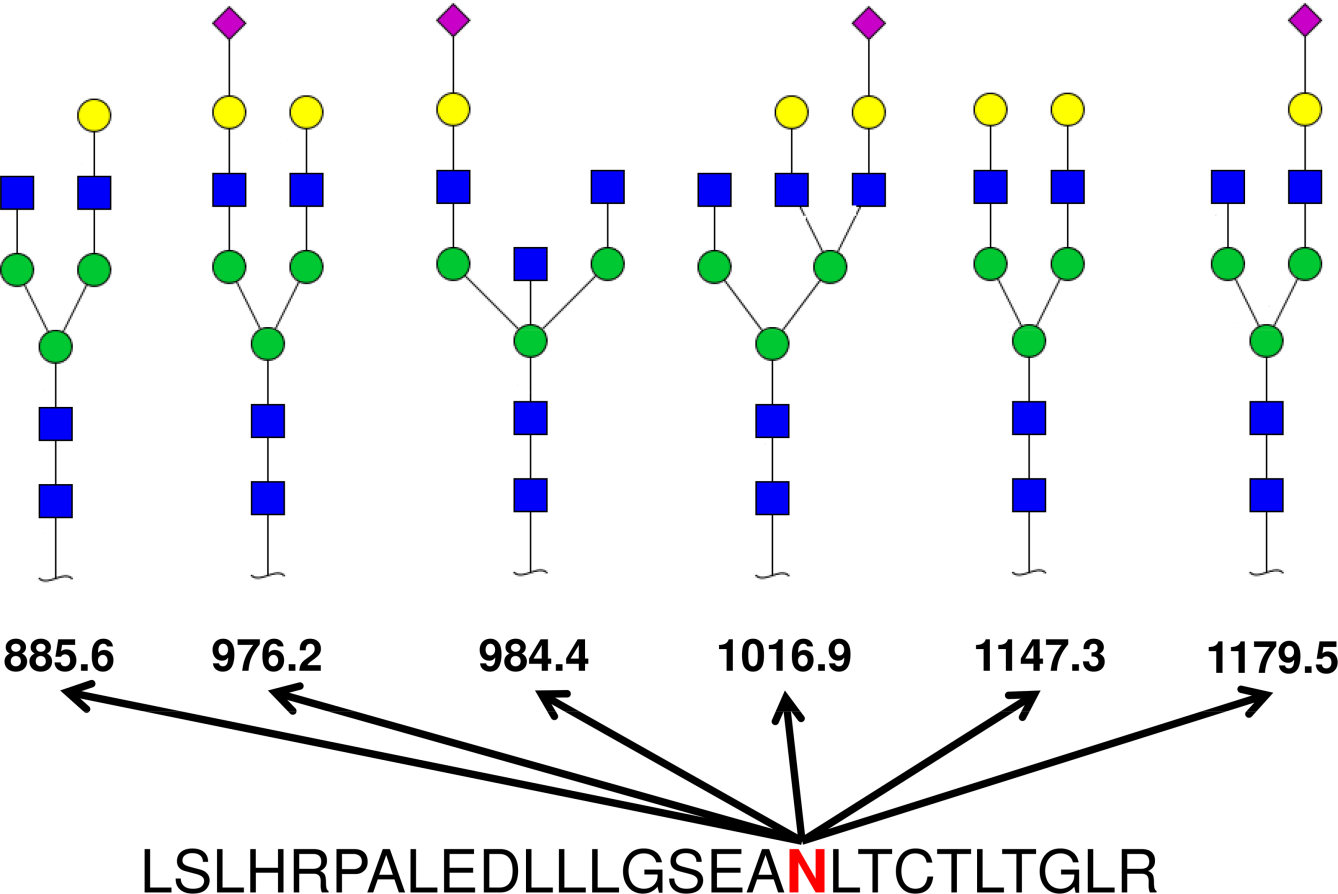
Structural features of representative examples of N-glycopeptides. An N-glycopeptide of immunoglobulin-A (IgA) heavy chain is shown here from which six different glycan compositions were found. Single peptide contained six different glycan compositions making six types of N-glycopeptides which were all identified from their respective spectrums. The spectrum of these N-glycopeptides matched to database entries (GlyycomeDB). These structural diagrams have the linkage specific information removed because it is not possible to infer linkage information (such as α- and β-glycosidic bonds) from the CID-MS/MS spectrum with currently used search tool. Blue squares are N-acetylglucosamines, green circles are mannoses, yellow circles are galactoses and purple rotated squares are N-acetylneuraminic acids (sialic acids).

Glycan scores of these 24 N-glycopeptides ranged from 6.21 to 50.66 while overall glycopeptide scores were found to be from 28.54 to 85.11. Out of the 24 glycopeptides which were identified by targeted N-glycoproteomics, nineteen glycan compositions were found to be sialylated. Twenty glycan structures for as many numbers of glycopeptides were proposed while 4 compositions were constructed *denovo*. For detailed description of how these compositions and structures are generated, see methods and our previous studies[14–17]. Twelve sialylated glycopeptides were bi-antennary as can be claimed by number of sialic acid as typically N-glycan branch is end capped with sialic acid and polysialic acid is very rare. Proposed structure S2H5N4 (S: Sialic acid, H: Hexose, N: HexNAc, Accession number: G90099GV, Glycome-DB) was found on five glycopeptides. One structure was found to be tri antennary (S3H6N5) while others were mono-sialylated mono or bi-antennary structures. Out of the four non-sialylated glycopeptides, one was constructed denovo, two were core fucosylated while one had a potential LacDiNAc motif. In total, six structures were proposed for fucosylated glycopeptides. Largest microheterogeneity was found for Immunoglboulin-A heavy chain. For IgA heavy chain peptide (LSLHRPALEDLLLGSEANLTCTLTGLR), six different glycan compositions were found attached to the same glycosylation site (Fig 4).

These glycan compositions and structures were obtained by matching to the database (GlycomeDB) but we still looked for confirmation of some features such as sialic acids manually. It was possible to confirm the sialic acid in all cases. All the annotated spectra are provided in S1 Supplementary Annotated Spectra.

### Further statistical analysis

**Fig 5:**
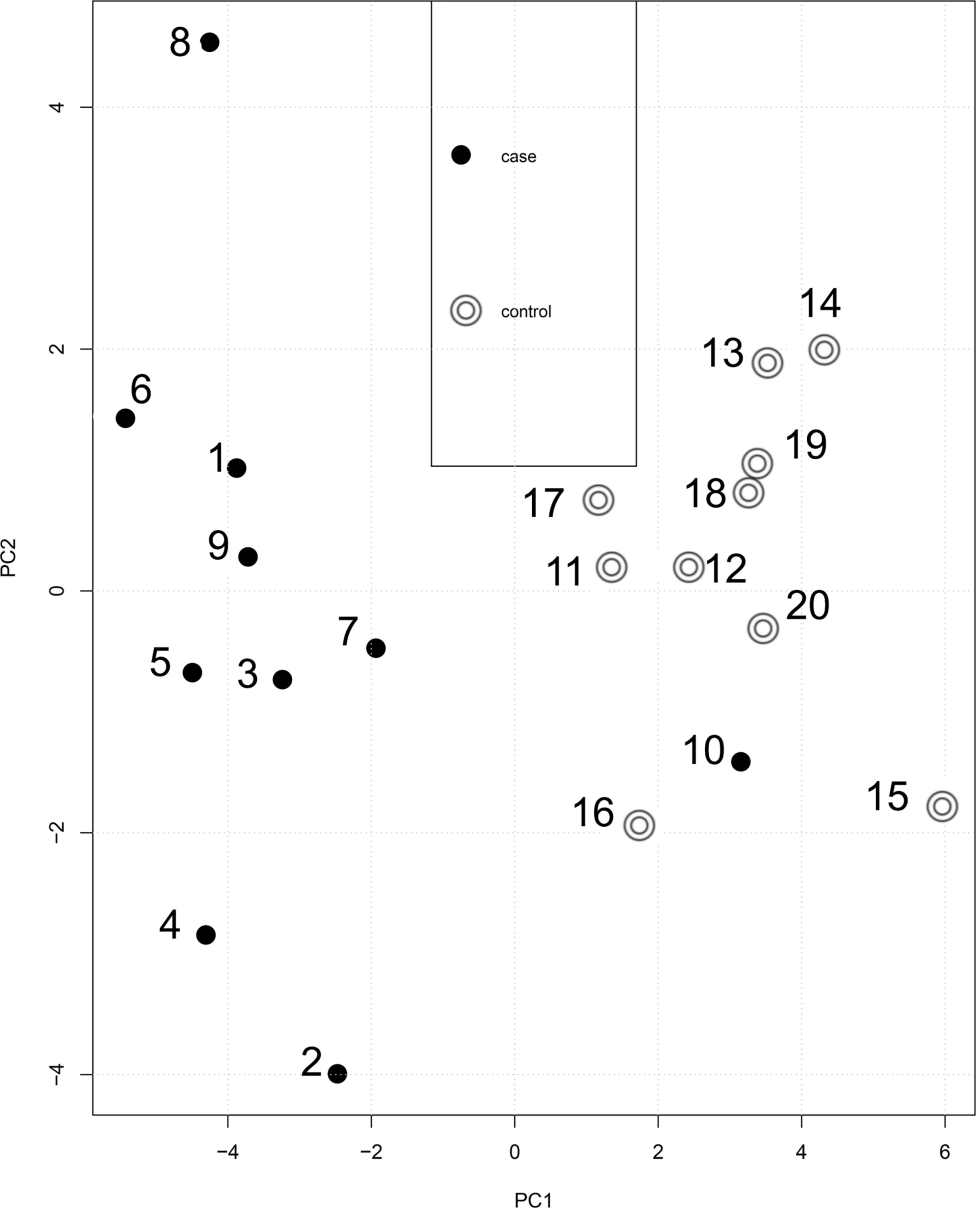
Principal component analysis. PCA was performed on only the N-glycopeptides ions which were identified and their peptide sequence, glycan composition and proposed structure was elucidated. This PCA gave us separation of the cases form controls. Principal component 1 (PC1) is on X-axis and principal component 2 (PC2) is on Y-axis. Red circles are controls and black circles are controls.

All glycopeptide ions quantified were used for self organizing maps (SOM) clustering as described in methods. This clustering gave all cases clustered into two classes at the ends of the clustered space while all controls were in between (Figure B in S1 File). One case sample (Case 10) was found to be mixing with the controls. We then extracted the data for the identified N-glycopeptides from the main table and used it to perform the PCA only with this data. The PCA for identified ions gave us superior separation of the cases and controls (Fig 5).

**Fig 6:**
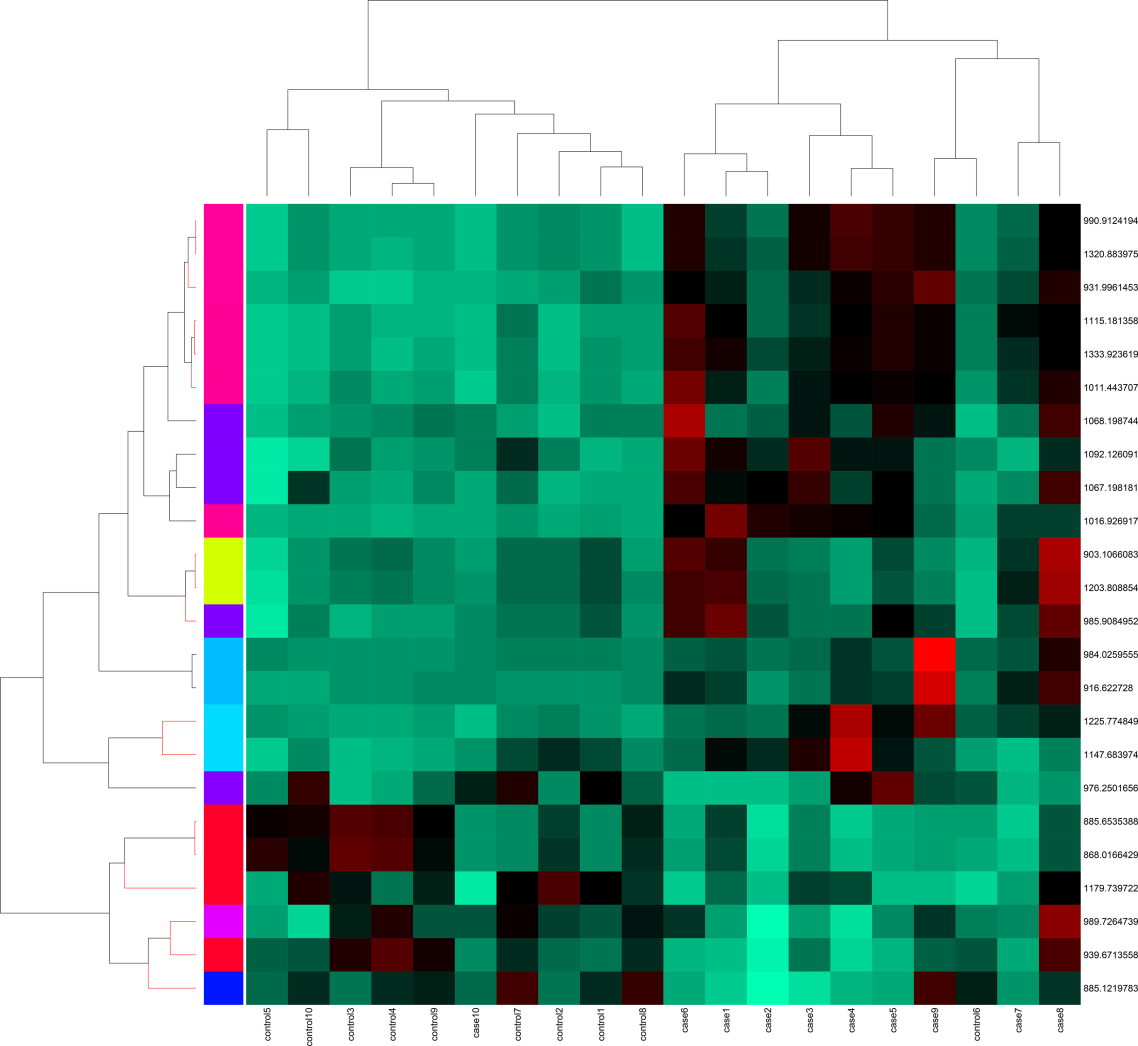
Self organizing map (SOM). SOM clustering of the 24 identified N-glycopeptide ions is presented in this figure.

We then used SOM clustering on the data of identified glycopeptides which is presented in Fig 6.

The SOM analysis for identified glycopeptides was able to cluster cases and controls separately and separation was near complete. Classical hierarchical clustering gave us similar results (Figure C in S1 File). However, S-Plot significant ions (Table B in S2 File) were able to best separate the cases and controls in PCA (Figure A in S1 File). We further calculated area under the curve values (AUC) for these identified 24 N-glycopeptides. Only 4 identified N-glycopeptides had AUC values less than 0.7, most others had AUC values of 0.8 and higher and some of them had AUC values of 0.9 or higher (Table 1).

## Discussion

Procalcitonin (PCT) alone, and also in combination with C-reactive protein (CRP) has been long recognized as a marker of the systemic inflammatory response to bacterial infections[4, 19]. However several studies have shown that PCT and CRP are not sensitive and/or specific enough to diagnose bacteremia or to differentiate it from other systemic inflammatory responses[7–9, 20]. Blood culture is still preferred to make a confirmed diagnosis, however several factors such as time needed to obtain results, false negative cases, and effect of pre-analytical variables on result outcomes limit its use[21–23].

Plasma glycoproteins have been shown to change during serious inflammation like sepsis in intensive care unit patients and in bacteremia[11, 24]. In this study we showed that several N-glycopeptides differed in bacteremic patients from control patients with non-bacteremic infections.

There are very early changes in glycosylation of proteins in host plasma and/or serum associated with inflammation and infection. It was shown that N-glycosylated receptors are involved in internalization of some bacterial species[25]. An evidence supported theory has been proposed that microbes manipulate the host glycosylation machinery to use it to their advantage[26]. These glycosylation changes can be detected and be used as early glyco-biomarkers if sufficiently sensitive methodologies of analysis are available. Proteomics can also identify biomarkers of bloodstream infection as we have previously shown[12] however, N-glycosylation is more sensitive to changes in physiology and it provides another dimension of selection for the biomarkers. It has been shown that changes in N-glycosylation determine the susceptibility to infection and influence the host response[27]. This implies that glyco-biomarkers of bloodstream infections will have the additional specificity compared to protein biomarkers which might be elevated in other conditions also. We have shown in the past that it is possible to quantify and identify intact N-glycopeptides with their peptide sequence, glycan composition and proposed structures[15–17]. In the current study, this technology was used in the setting where blood culture positive and negative patients were analyzed for their N-glycopeptide content. We have quantified 368 N-glycopeptide ions from plasma of these patients and subsequently identified 24 glycopeptides comprehensively for their peptide sequence, glycan composition and glycan structures in a targeted manner. These 24 confidently identified N-glycopeptide belonged to 8 different glycoproteins which are alpha-1-antitryspin, ceruloplasmin, Chondroitin sulfate N-acetylgalactosaminyltransferase 1 (CGAT1), hemopexin, haptoglobin, haptoglobin-related protein and heavy chains of immunoglobulin A and G. For seven out of these 8 proteins, excluding only CGAT1, we compared the protein levels among the cases and controls from our previous proteomics study and we found that none of them were statistically significantly different among cases and controls [12]. However, their N-glycopeptides expression levels were statistically significantly different among cases and controls (in current study) suggesting that N-glycosylation of these proteins is changing between cases and controls. This also suggests that plasma N-glycoproteomics has the power to provide novel and different markers of the systemic diseases even when they are not captured by proteomics alone. These markers can also potentially be more sensitive to early changes.

A previous study has identified major glycoproteins from plasma samples of sepsis patients and linked them to the survival status of patients[11]. In this study, N-glycopeptides were enriched by hydrazide chemistry and released from the matrix by PNGAse-F therefore no information about glycan composition and/or structure was obtained. Our study reports the complete information about 24 N-glycopeptides which are shown to change between blood culture positive and negative status of patients by various statistical methods. These peptides have been previously identified in sepsis patients[11] other than one peptide of CGAT1 (m/z 1067.198) which was shown to increase 1.8 fold in blood culture positive patients compared to blood culture negative ones (Table A in S2 File). We have identified that on one peptide of IgA1, six different type of complex type N-glycans are present on the same site suggesting moderate amount of microheterogeinity. Three of these N-glycopeptides were present in higher amounts in blood culture positive patients’ plasma while 3 others had reduced levels in them (Table A in S2 File). Out of these six N-glycoepptides 4 were sialylated and 2 were non-sialylated complex type glycans. It is known that patients with an automimmune inflammatory disorder (Sjögren’s syndrome) have higher levels of sialylated N-glycans on IgA1[28]. Increased sialic acid levels are known to be present during bacterial and viral infections and these levels support the growth of the microbes[29]. Other glycoproteins identified in our study were acute phase proteins such as hemopexin, alpha-1-antitrypsin, ceruloplasmin and haptoglobin. Glycosylation of acute phase proteins changes rapidly in host response to inflammation and infection[30–32]. However, most of these identified acute phase N-glycopeptides seem to be upregulated in plasma of blood culture positive patients compared to febrile controls (blood culture negative). Further studies on these N-glycopeptides are warranted to correlate their levels to prognosis of bloodstream infected patients and to the blood culture result outcomes.

Using t-test, these identified glycopeptide levels were significantly different among cases and controls (Table A in S2 File). Moreover, our panel of identified glycopeptides can separate the blood culture positive versus negative febrile patients at a very early stage, using PCA and SOM clustering. Hierarchical clustering also gave similar results. This is a statistical validation of our results that supervised and unsupervised statistical methods using different algorithms gave similar results. If we consider only S-plot significant glycopeptides, then SOM clustering was able to separate the two classes. The reason for us to do so many different analyses was to check the robustness of the results. Various different methods use different algorithms and if a result holds true in many of them then its serves as validation of the results. We had also selected multiple other glycopeptides (149 total including S-plot ions) for identification based on t-test p values and intensity threshold in samples. We have identified 24 glycopeptide with high confidence from these 149 ions. This has to be noted that in MS/MS identification of glycopeptides requires very high quality spectrum to be interpreted by the specialized search engines (GlycopeptideId in our case). Low abundant glycopeptides as a result of low amounts or microheterogeneity are particularly difficult to identify by currently available technology even though its state-of-the-art. Nevertheless, these 24 identified glycopeptides are able to separate the cases from controls by various statistical methods. Moreover, PCA analysis of S-Plot ions was able to separate cases from controls (Figure A in S1 File). Cases were spread more in PCA space compared to controls which highlights that there is some degree of heterogeneity in the Cases, in terms of the levels of N-glycopeptides. Early bacteremia cases can be reliably separated from controls using plasma N-glycopeptide expression levels. This separation supports and validates our decision to select these 2 groups in the first place and shows promising clinical utility. It has to be emphasized that these results hold true in a bacterial strain independent manner.

We analyzed the identified glycopeptides by ROC curve analysis to see how they could separate bacteremic cases from noin-bacteremic controls which gave us different values of area under the curve (AUC) values (Table 1) for different glycopeptides. The highest AUC of 0.954 was found for the N-glycopeptide (m/z 1016.927) of IgA heavy chain which had the composition of S1H5N5. Glycosylation of IgA has been known to affect the effector functions of IgA[33] and it’s only logical that it was increased 5.27 times in confirmed cases of bloodstream infection. It also had a good p(Corr) value of -0.76 in OPLS-DA S-Plot analysis. Specific hybrid ELISA test measuring the N-glycosylation of IgA using dual-approach (antibody to capture IgA and lectins to measure N-glycosylation) could be potentially developed in the future to aid in clinical diagnosis.

In summary, all of the identified N-glycopeptides were significantly different between the 2 groups by t-test and many of them had good ROC values (Table 1). Supervised and unsupervised clustering was also able to separate the cases form controls. Ions found to be significant in S-Plot, ten of which were identified, were able to completely separate the cases and controls in various statistical analyses. These potential glycopeptide ions (even the ones quantified but not identified in the current study) are particularly important as they can separate bloodstream infection from the febrile controls. More importantly, these glycopeptide ions were observed in bacteremic patients due a variety of different bacteria. We have chosen to do a targeted analysis as it is well suited to be utilized rapidly by other groups also and future studies can provide extra layer of validation without the need to identify the N-glycopeptides, which is technically very challenging. A stringent statistical analysis combined with quantitative N-glycoproteomics is a good combination (with enough sensitivity and specificity) which can provide important clinical biomarkers for bloodstream infections which are strain-independent and therefore clinically more important.

## Methods

### Patient samples

Patient samples were handled as described in our previous study[12]. Briefly, blood samples were collected to lithium-heparin tubes from adult febrile patients (age range 53–91, median age 76 years) coming to the outpatient emergency clinic of Peijas Hospital, Helsinki University Hospital (Vantaa, Finland). Blood cultures were taken based on clinical suspicion of a serious infection both from the later confirmed cases and from the febrile controls on the same grounds. An approval for the study was received from the Ethics Committee of Medical Sciences (HUS 169/13/01/2014) and a written informed consent was obtained from all subjects at the time of plasma sample collection. Blood culturing was performed by using BacT/ALERT® FA Plus and BacT/ALERT® FN Plus blood culture bottles (BioMerieux, Durham, NC, USA) and BacT ALERT 3D incubator (BioMerieux). Identification of bacteria in positive blood cultures was done by Vitek MS MALDI-TOF instrument (bioMerieux). For proteomic analyses blood samples (3–5 ml) were adjusted to room temperature for 15 min. Subsequently, plasma was separated by centrifugation (1200xg) for 10 minutes at room temperature. Plasma samples were stored at -70°C until tested at the same time.

### Trypsin digestion

The complete protocol used in the study has been submitted to the protocols.io with the following doi (10.17504/protocols.io.ncadase). Albumin was removed from plasma samples according to manufacturer’s instructions (Pierce, Thermo Fisher). BCA assay was used for protein concentration measurement. After drying, plasma sample volume equivalent to 350μg of protein, 7μg of bovine Fetuin protein was added and samples were trypsin digested as described previously[16]. Briefly, 10mM DTT (final concentration) was added to the samples and incubated for 1 Hour at RT with shaking. Subsequently, 40mM (final concentration) of Iodoacetamide was added and samples were again incubated at RT for 1 hour with shaking in dark. 40mM DTT was further added to avoid overalkylation by Iodoacetamide. Seven μg of bovine pancreatic trypsin was added to the solution and incubated at 37°C overnight. 60 μL of tryptic peptides were diluted by 540 μL of 10mM HEPES buffer pH 7.4 containing 1mM CaCl2 and 1mM MnCl2. this 600 μL mixture was applied to lectin-agarose columns. Briefly, Con-A: SNA: LCA: AAL were used in ratio of 5:3:3:1 for a final volume of lectin resin slurry of 150 μL. micro-columns containing lectin resins mixed with samples were incubated at 4°C on rotation overnight. Next day, three washes with HEPES buffer was performed and N-glycopeptides were eluted with sugar-mix solution containing fucose, α-methyl mannoside, α-methyl glucoside and lactose followed by second elution with 1% formic acid. N-glycopeptides were cleaned by C18 micro-columns according to manufacturer’s instructions. Resulting N-glycopeptides were dissolved in 0.1% formic acid before being analyzed by UPLC-MS.

### UPLC-MS

A Waters SYNAPT G2 High Definition MS connected to a Waters nanoACQUITY UPLC was used for the analysis. Positive mode with sensitivity mode was used for MSE (100–2000 Da mass range) and FAST DDA (positive and sensitivity) mode for N-glycopeptide fragmentation (50–2500 Da mass range). For MSE, the scan time was 1 second and trap collision energy (high energy function) was ramped from 14 to 44 V. For FAST DDA experiment, the scan time was 1 second for MS and MS/MS. Continuum data format and deisotope peak selection was applied. No exclusion criterion was applied and for inclusion, targeted N-glycopeptide m/z values were supplied as include files. Mass window for peak inclusion was 250 mDa and retention time window was 10 seconds. Trap collision energy was applied with low mass to high mass ramp from 20 to 60 V. Calibration was performed with sodium formate. The trapping column was a nanoACQUITY UPLC Trap, 180 μm x 20 mm (5 μm), Symmetry®C18, and the analytical column was a nanoACQUITY UPLC, 75 μm x 100 mm (1.8 μm), HSS T3. Samples were loaded, trapped and washed for two minutes with 8.0 μL/min with 1% B. The analytical gradient used is as follows: 0–1 minutes 1% B, at 2 minutes 5% B, at 45 minutes 30% B, at 48 minutes 50% B, at 50 minutes 85% B, at 53 minutes 85% B, at 54 minutes 1% B and at 60 minutes 1% B with 450nL/min for MSE while 300 nL/min for N-glycopeptide fragmentation.

### Data analysis

The raw files were imported to Progenesis QI for proteomics software (Version V2, Nonlinear Dynamics, Newcastle, UK) using lock mass correction with 785.8426 m/z, corresponding to doubly charged Glu1-Fibrinopeptide B. Default parameters for peak picking and alignment algorithm were used. The software facilitated the label-free quantification. Known amount of bovine Fetuin added to the samples before trypsin digestion allowed the normalization of N-glycopeptides by known bovine Fetuin N-glycopeptides. Briefly, the Progenesis QI for Proteomics software functions in the following way

#### Run alignment

The run with the most features (ions) is used as reference and all other runs are aligned to the reference.

#### Peak picking

An aggregate data set is created from the aligned runs, which contains all peak information from all sample files. This aggregate peak list is then matched to each sample.

#### Ion abundance quantification

Peptide ion abundance is a sum of areas which is calculated using intensities of the peaks of a peptide ion's and peaks' width.

Subsequent to these steps a filter is applied and we select +3 to +5 charged ions as potential N-glycopeptides. Thereafter, the groups are compared to show differences in cases vs controls and as a last step, MS/MS is performed to identify the N-glycopeptides.

N-glycopeptide ions were identified as previously described[15]. Briefly, the MS/MS spectra were deconvoluted in MaxEnt3 module of Waters MassLynx 4.1 software and saved as peak lists (.pkl). Identification of N-glycopeptides was performed on the publicly available software GlycopeptideID, which can perform automated CID MS/MS spectrum analysis. The principle of this method is explained in detail in our previous publications[15–17]. Briefly, a database of tryptic peptides (Uniprot, 2 misscleavges allowed, mandatory to have NXS/T/C, P! in the peptide sequence, where X is any amino acid other than proline) from known human proteins is used, and the combined and deconvoluted MS/MS spectra are imported as .pkl files. Software matches the MS2 spectra against a peptide database (specified above) and scores potential peptide backbones. Next, it searches glycan compositions against a glycan database and returns glycan compositions which are fitted onto the spectrum (glycan score). The glycopeptide score is the sum of the peptide and glycan scores. The results are ranked, and for each potential result, an annotated spectrum is drawn for manual assessment of the matching y and b ions, glycan fragments and glycopeptide fragments. The target-decoy search approach is employed to calculate false-discovery rate (FDR). The decoy database was generating by reversing the peptide sequences of tryptic peptide database specified above.

The GlycopeptideId search algorithm has two main steps: i) scoring possible peptide backbones by matching the MS2 spectra against a peptide database, and ii) scoring the MS2 spectra against the glycans which match to the precursor–peptide mass difference. The outcome is a list of the best matching glycopeptides for each precursor. The results are ordered by the total score which is related to the (binomial) probability that a similar match could be achieved by random sampling. Score to a theoretical glycopeptide is defined as a negative logarithm of a probability that the measured spectrum will have equal or more shared peaks with a random set of fragments. The probability PR that the random spectrum R has more or equal shared peaks than the glycopeptide spectrum G is integrated into the formula as Score = −log(PR). The score defined here is related to the concept of “Ascore” which has been used for phosphorylation site determination [34].

GlycopeptideID gives glycan compositions as one-letter abbreviations H: Hexose, N: Hexosamine, S: Sialic acid, and F: Fucose, and the number following indicates the amount of the monosaccharides. Accordingly, S2H6N5 stands for a glycan containing two sialic acids, six hexoses, and five hexosamines. The glycan structure output format in the GlycopeptideID is a simplified version of the consortium for functional genomics (CFG) Modified IUPAC condensed format (www.functionalglycomics.org/static/consortium/Nomenclature.shtml). The stereoisomer (α, β) and the regioisomer (for ex. 1–4) notations are omitted and the long monosaccharide names are replaced by single letter codes (H:Hex, N:HexNAc, F:Fuc, S:NeuAc). This format consists of linear glycan sequences with branching shown by parenthesis and written from nonreducing to reducing end. As an example a core fucosylated, complex N-glycan with two branches shown in CFG format as NeuAcα 2-6Gal β 1-4GlcNAc β 1-2Man α 1-6(NeuAcα 2-6Gal β 1-4GlcNAc β 1-2Man α 1–3)Manβ 1-4GlcNAc β 1-4(Fucα 1–6)GlcNAc-Asn is written as SHNH(SHNH)HN(F)N. This format is simpler and has a compact notation and it only shows that information which can be identified with the current experimental technology. for exmaple it does not say anything about the nature of the glycosidic bond (α or β).

### Statistical analysis

Principal component analysis was done in three different softwares namely, Progenesis, R programming and PAST3.0. Orthogonal projections to latent structures-discriminant analysis (OPLS-DA) modeling, which generated the S-Plot was performed in EZInfo 3.0. In R, PCA was performed on scaled data using prcomp R function. Self-Organizing map clustering was performed on data consists of X number of samples using R package SOM with parameters (.xdim = 5, ydim = 6, topol = "hexa", neigh = "gaussian") Before performing clustering, data was centered and scaled. SOM provides better visualisation of complex data and is robust enough to tackle minor experimental variation. Subsequently heatmap was generated using parameters from hclust function in R. Hierarchical clustering based dendrogram was generated in PAST3.0.

"The mass spectrometry N-glycoproteomics data have been deposited to the ProteomeXchange Consortium via the PRIDE [35] partner repository with the dataset identifier PXD009048".

## Supporting information

S1 File **This file contains supplementary Figures A-C.** Figure A shows N-glycopeptides ions found to be significantly different between the 2 patients classes were used for running principal component analysis using the publicly available software PAST3.0. 1b-10b are cases while 11b-20b are controls. Figure B shows Self organizing maps clustering performed on all quantified potential N-glycopeptide ions. Cases and controls can be seen with magnification at the bottom of the figures. Figure C shows classical hierarchical clustering based on only the identified N-glycopeptide ions. Triplicate run values were used which also show that all triplicates cluster together for each sample indicating superior chromatographic alignment.
(PDF)

S2 File This file contains Table A with all the quantified potential N-glycopeptide ions and Table B with significantly different potential N-glycopeptide ions found by Orthogonal projections to latent structures-discriminant analysis visualized by S-Plot.
(XLSX)

S1 Supplementary Annotated Spectra Annotated spectra for all identified N-glycopeptide ions. (PDF)
