# Supplementary figures and images for: Quantitative N-glycoproteomics reveals altered glycosylation levels of various plasma proteins in bloodstream infected patients
Source: PLoS One. 2018 Mar 29;13(3):e0195006. doi: 10.1371/journal.pone.0195006 (PMC5875812; doi:10.1371/journal.pone.0195006)

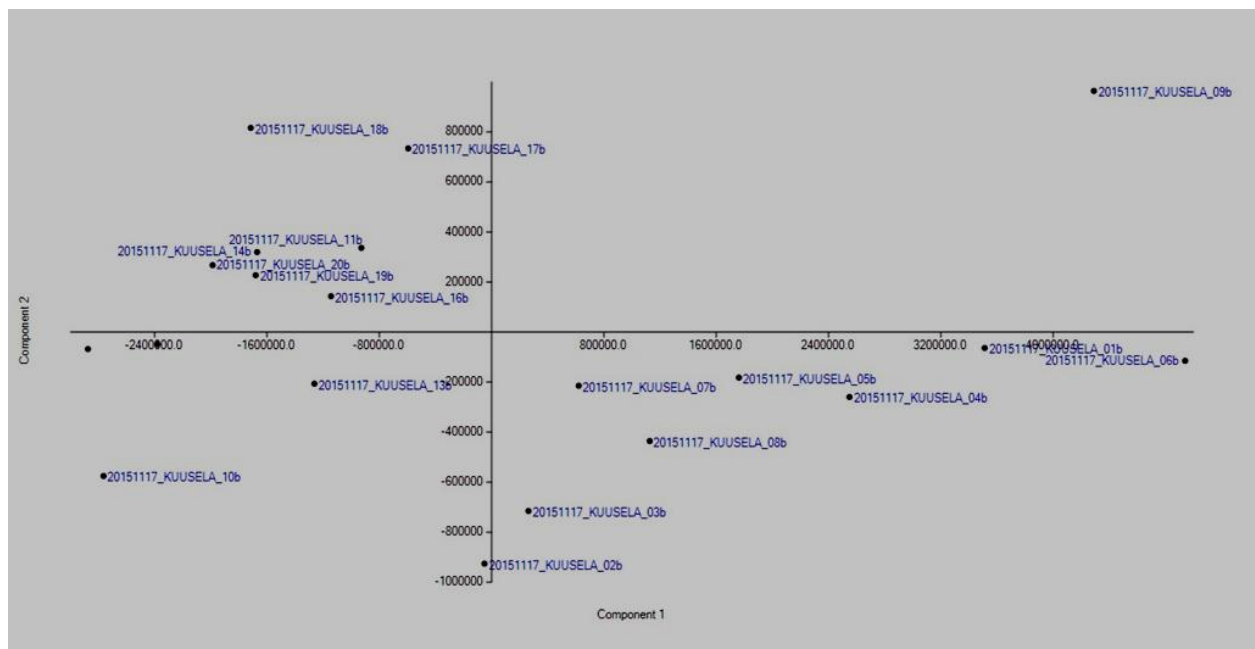

Figure A

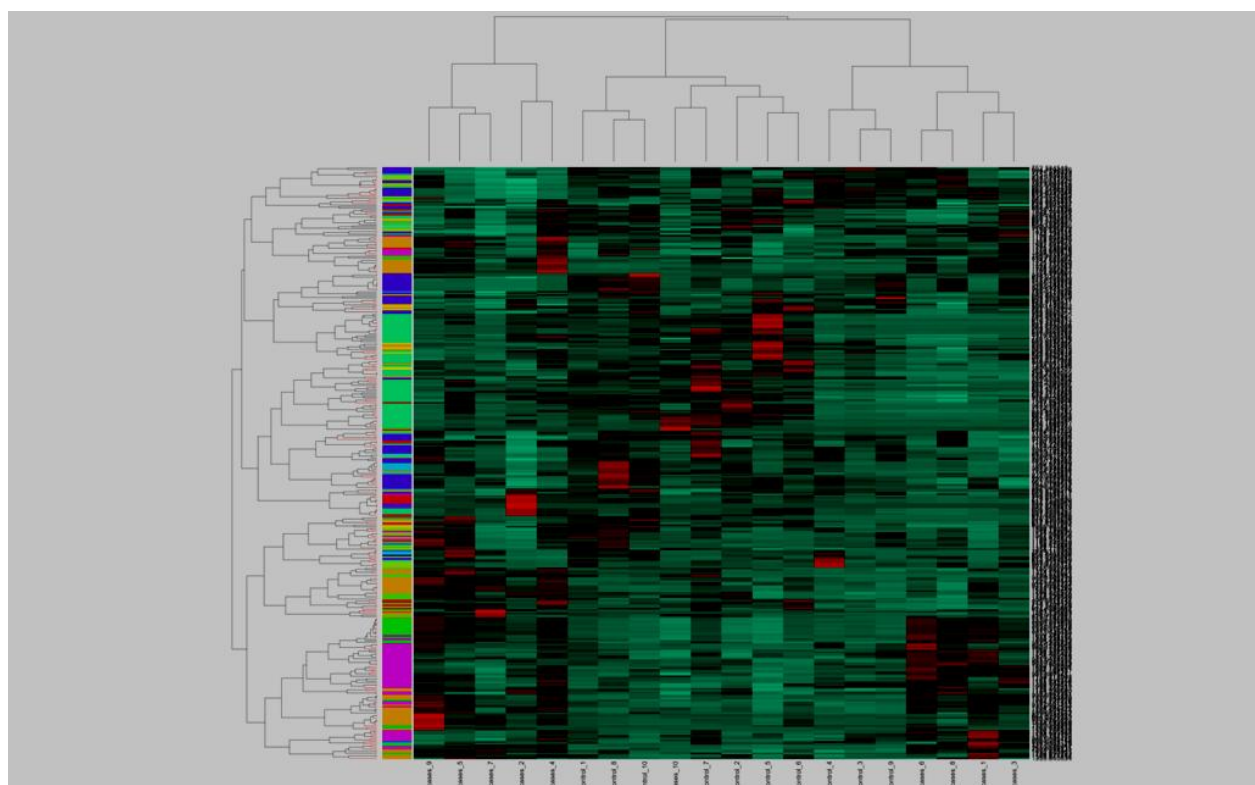

Figure B

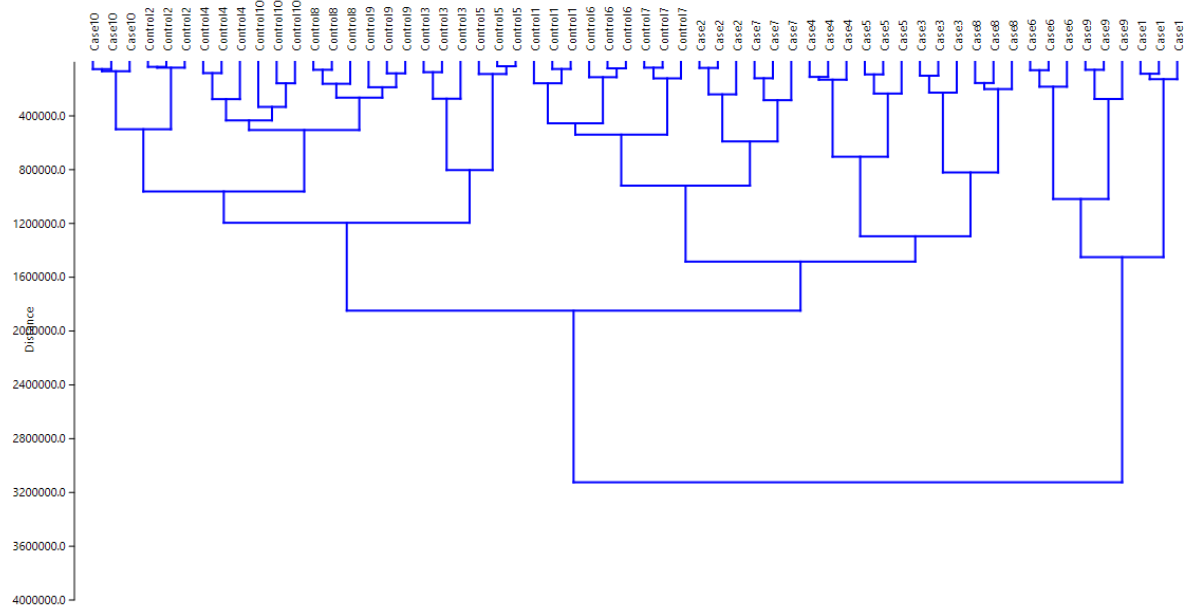

Figure C

Supplement: S1 File — This file contains supplementary Figures A-C. Figure A shows N-glycopeptides ions found to be significantly different between the 2 patients classes were used for running principal component analysis using the publicly available software PAST3.0. 1b-10b are cases while 11b-20b are controls. Figure B shows Self organizing maps clustering performed on all quantified potential N-glycopeptide ions. Cases and controls can be seen with magnification at the bottom of the figures. Figure C shows classical hierarchical clustering based on only the identified N-glycopeptide ions. Triplicate run values were used which also show that all triplicates cluster together for each sample indicating superior chromatographic alignment. (PDF) [file pone.0195006.s001.pdf]
