# Supplementary material for: Quantitative N-glycoproteomics reveals altered glycosylation levels of various plasma proteins in bloodstream infected patients
Source: PLoS One. 2018 Mar 29;13(3):e0195006. doi: 10.1371/journal.pone.0195006 (PMC5875812; doi:10.1371/journal.pone.0195006)
Supplement: S1 Supplementary Annotated Spectra — (PDF) [file pone.0195006.s003.pdf]

m/z 990.9476 charge 4 scan 0−0

Score= 77.59 , Hits= 53 , Explained Intensity= 0.45

Peptide: A1AT_HUMAN[268,283]:YLGNATAIFFLPDEGK

Glycan: SHNH(SHNH)HNN, S2H5N4

Charge: 4H

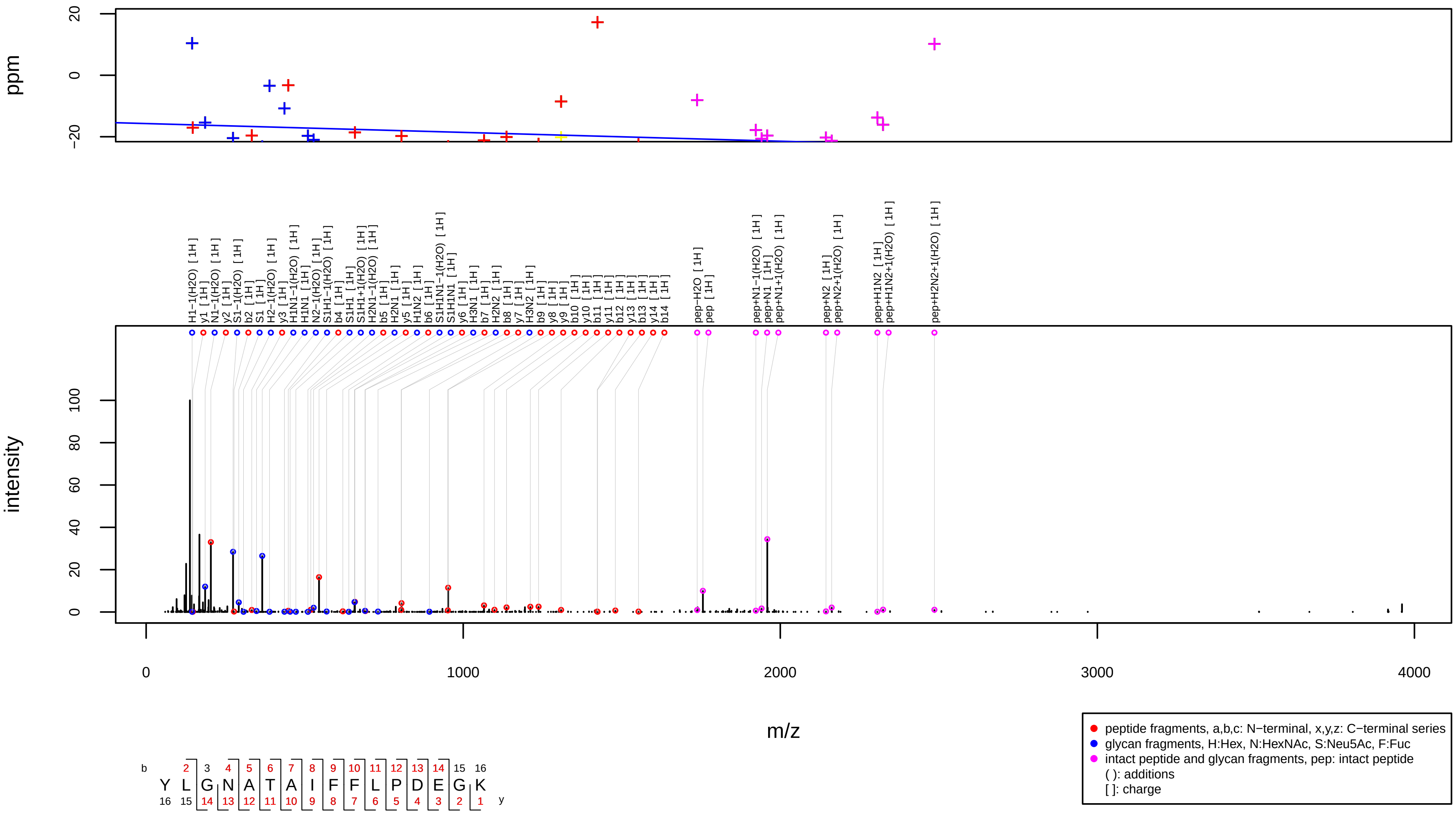

Glycopeptide ID − *Thu Mar 9 13:19:20 2017*

m/z 1011.4318 charge 4 scan 0−0

Score= 44.72 , Hits= 37 , Explained Intensity= 0.21

Peptide: A1AT_HUMAN[268,283]:YLGNATAIFFLPDEGK

Glycan: S2H3N6

Charge: 4H

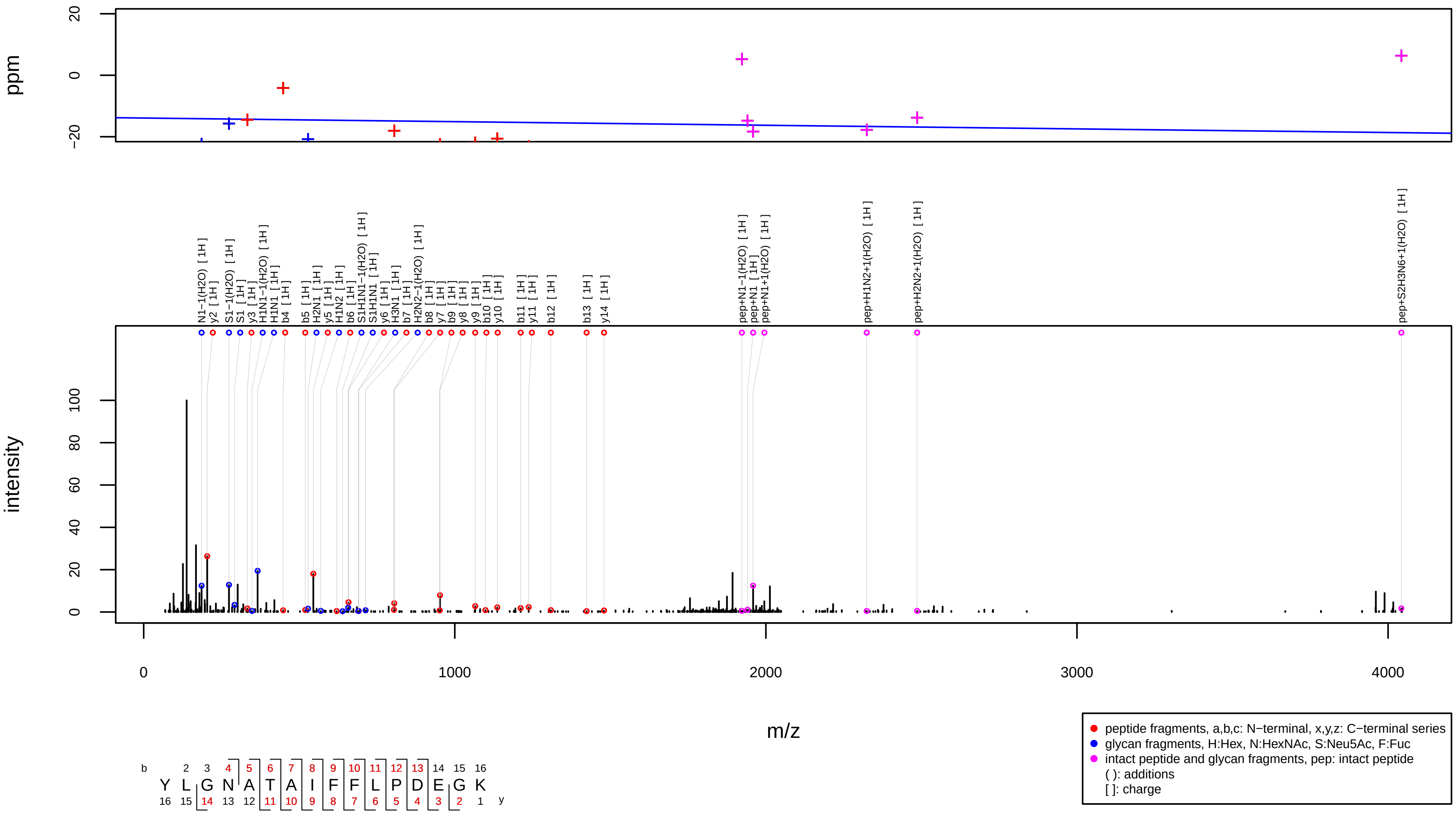

Glycopeptide ID − *Thu Mar 9 13:19:26 2017*

m/z 1320.927 charge 3 scan 0−0

Score= 70.66 , Hits= 48 , Explained Intensity= 0.47

Peptide: A1AT_HUMAN[268,283]:YLGNATAIFFLPDEGK

Glycan: SHNH(SHNH)HNN, S2H5N4

Charge: 3H

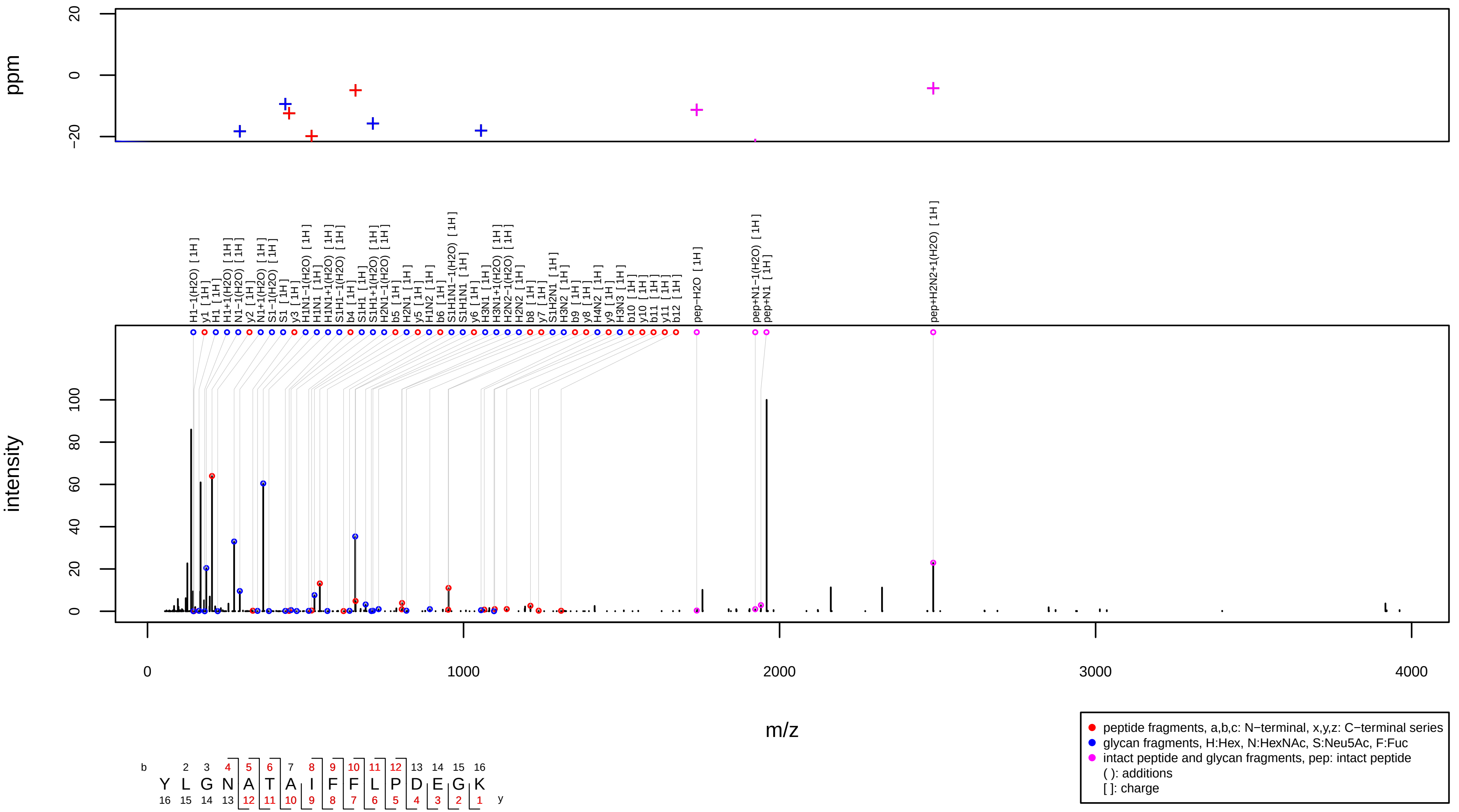

b		2	3	4	5	6	7	8	9	10	11	12	13	14	15	16	
	Y	L	G	N	A	T	A	I	F	F	L	P	D	E	G	K	
	16	15	14	13	12	11	10	9	8	7	6	5	4	3	2	1	y

Glycopeptide ID − *Thu Mar 9 13:19:24 2017*

m/z 1068.4491 charge 4 scan 0−0

Score= 40.55 , Hits= 36 , Explained Intensity= 0.2

Peptide: CERU_HUMAN[129,144]:EHEGAIYPDNTTDFQR

Glycan: H5N7F1

Charge: 4H

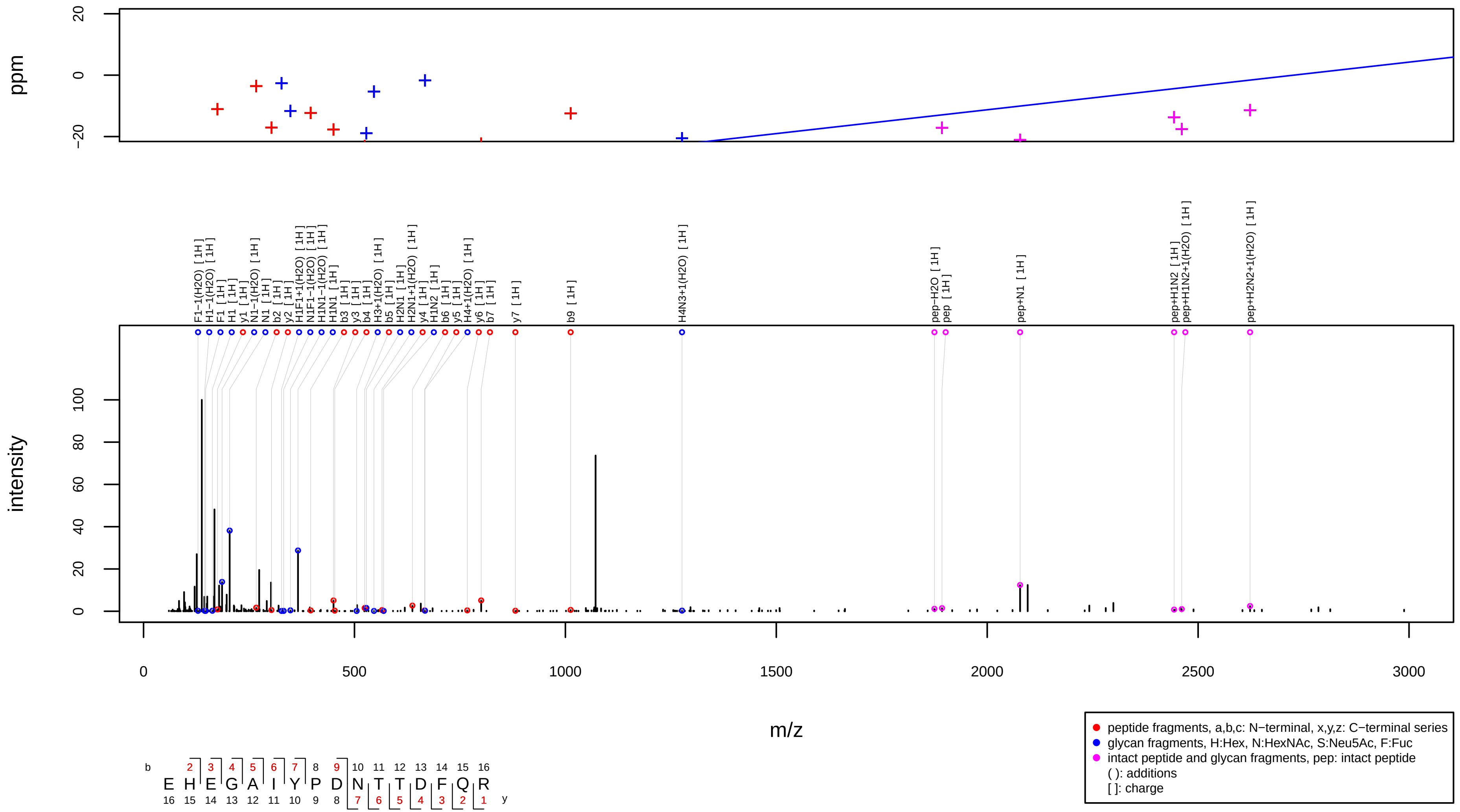

Glycopeptide ID − *Thu Mar 9 13:19:31 2017*

m/z 1067.238 charge 5 scan 0−0

Score= 28.54 , Hits= 33 , Explained Intensity= 0.26

Peptide: CGAT1_HUMAN[319,338]:AANFRNFTFIQLNGEFSRGK

Glycan: S2H10N4

Charge: 5H

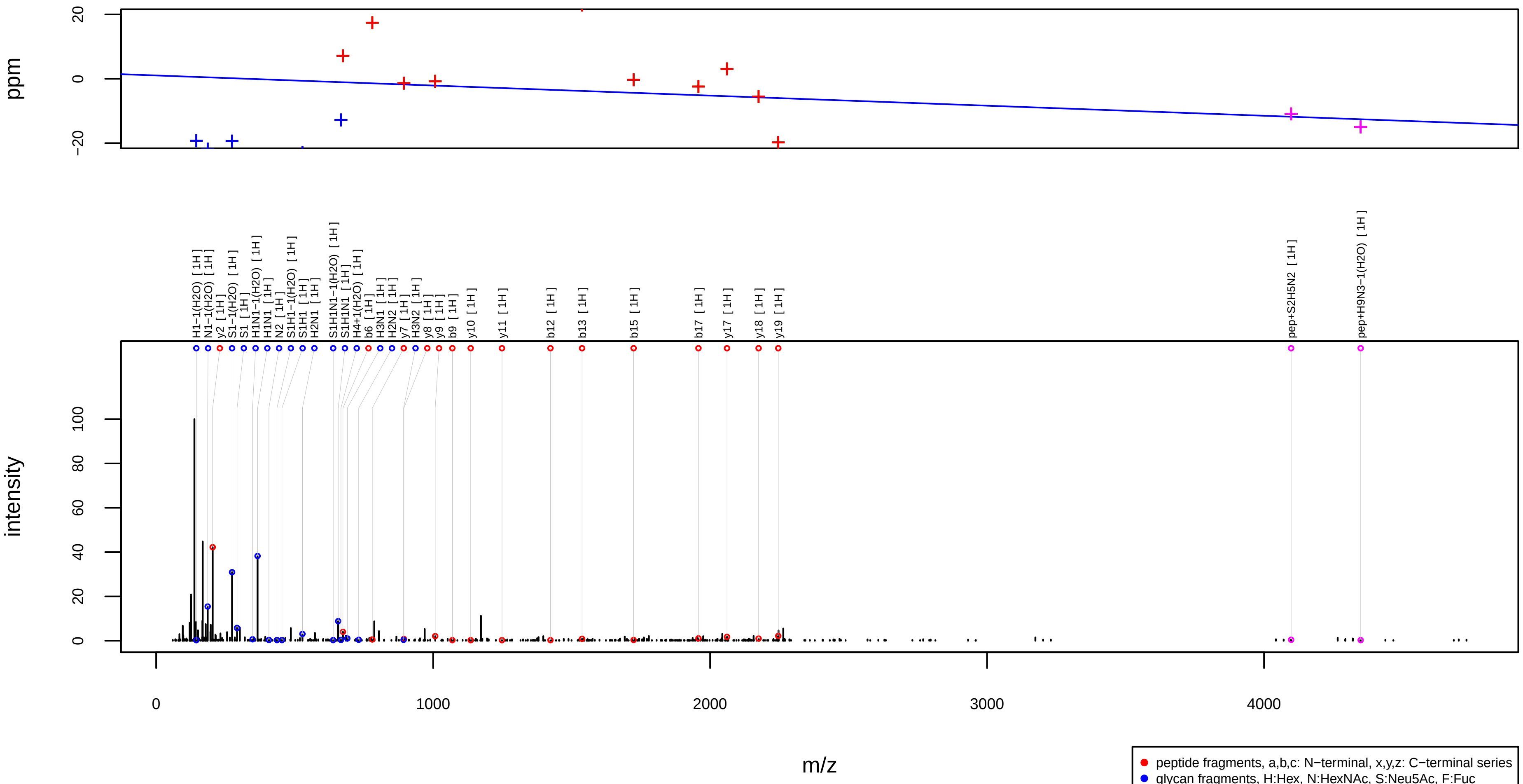

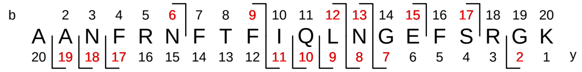

Glycopeptide ID − *Thu Mar 9 13:19:42 2017*

m/z 903.1394 charge 4 scan 0−0

Score= 51.02 , Hits= 40 , Explained Intensity= 0.33

Peptide: HEMO_HUMAN[181,193]:SWPAVGNCSSALR

Glycan: SHNH(SHNH)HNN, S2H5N4

Charge: 4H

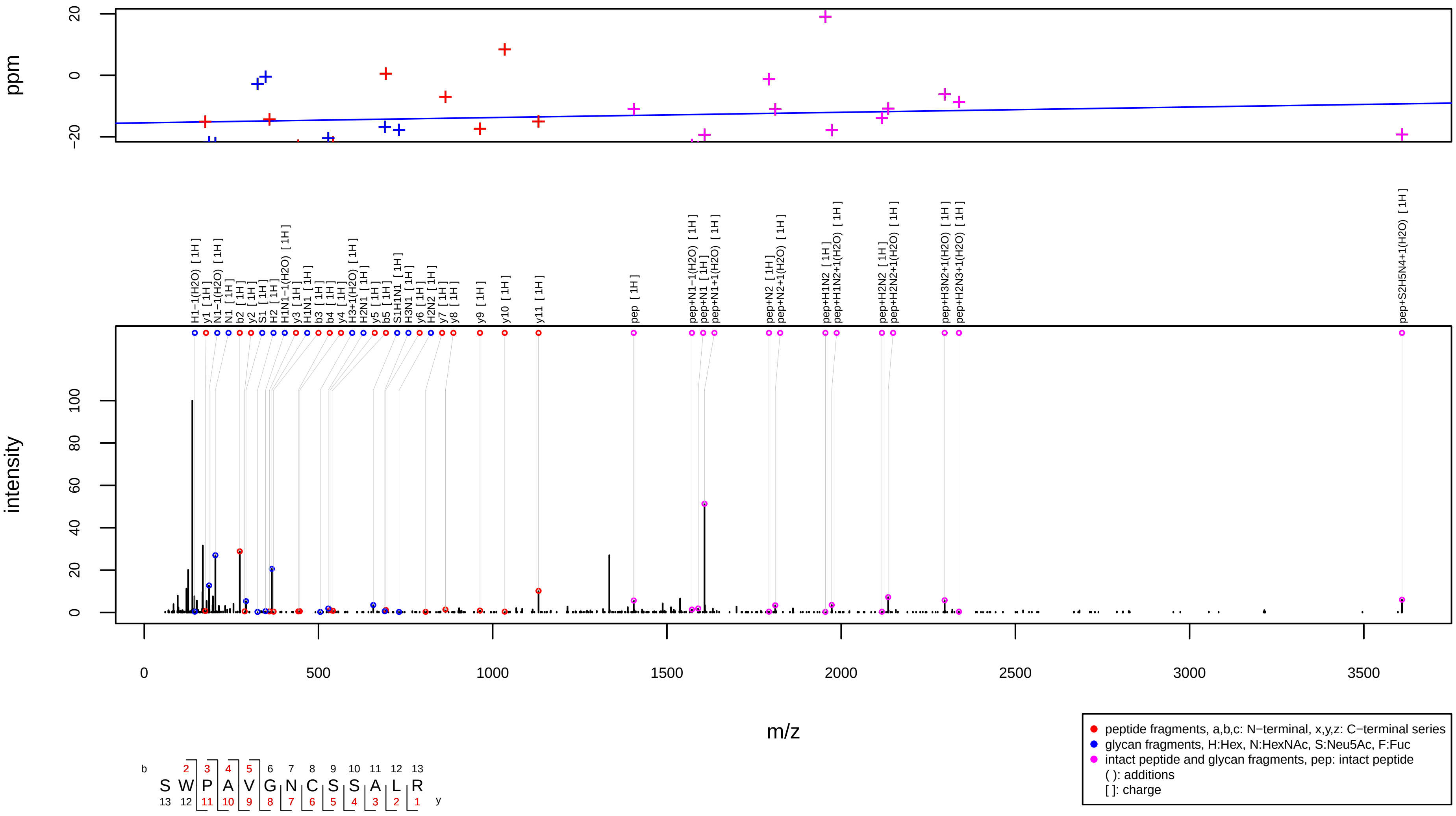

Glycopeptide ID − *Thu Mar 9 13:19:34 2017*

m/z 939.653 charge 4 scan 0−0

Score= 46.03 , Hits= 40 , Explained Intensity= 0.22

Peptide: HEMO_HUMAN[181,193]:SWPAVGNCSSALR
Glycan: SHNH(SHNH)HN(F)N, S2H5N4F1
Charge: 4H

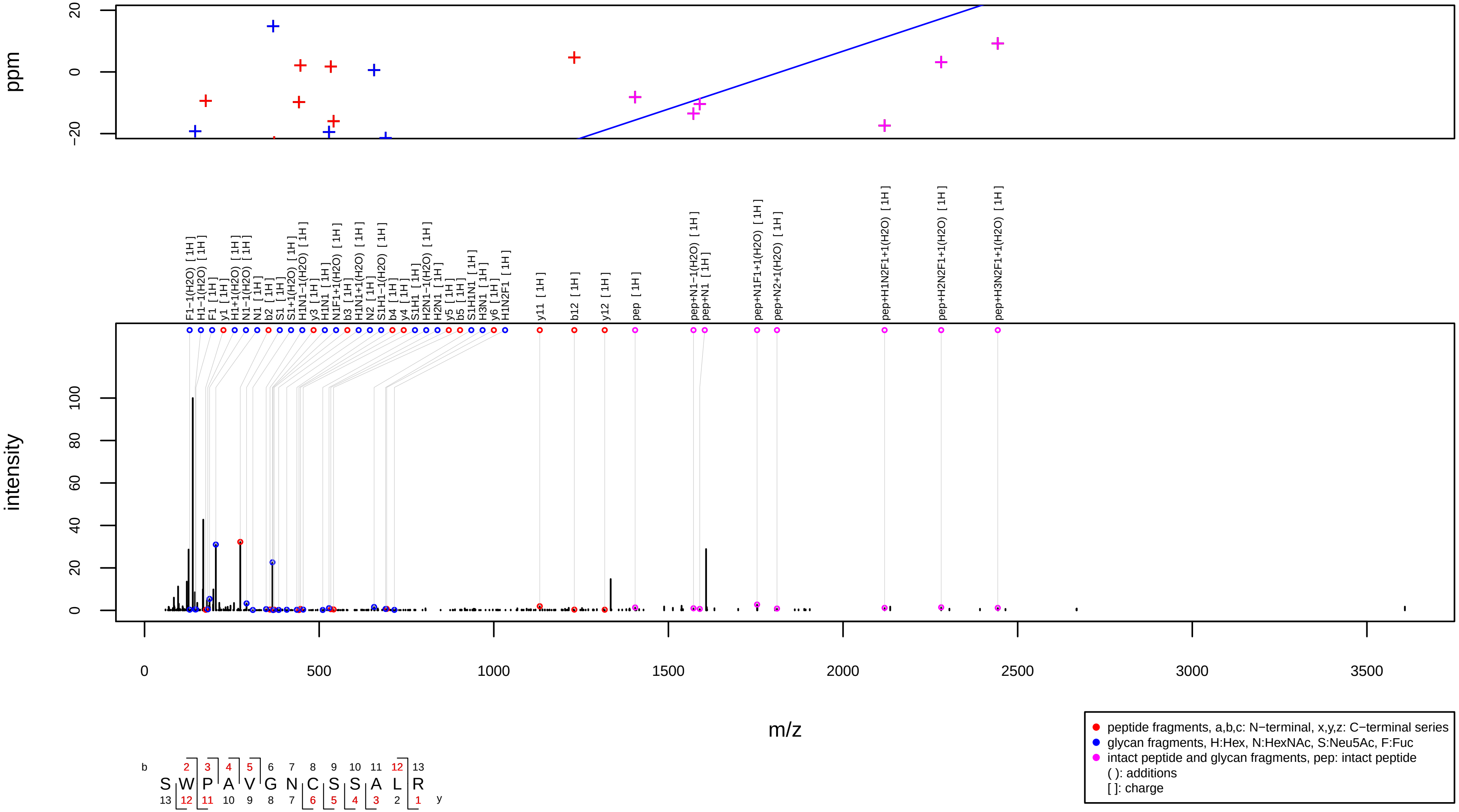

m/z 985.9345 charge 4 scan 0−0

Score= 39.93 , Hits= 27 , Explained Intensity= 0.25

Peptide: HEMO_HUMAN[447,462]:ALPQPQNVTSLLGCTH

Glycan: SHNH(SHNH)HNN, S2H5N4

Charge: 4H

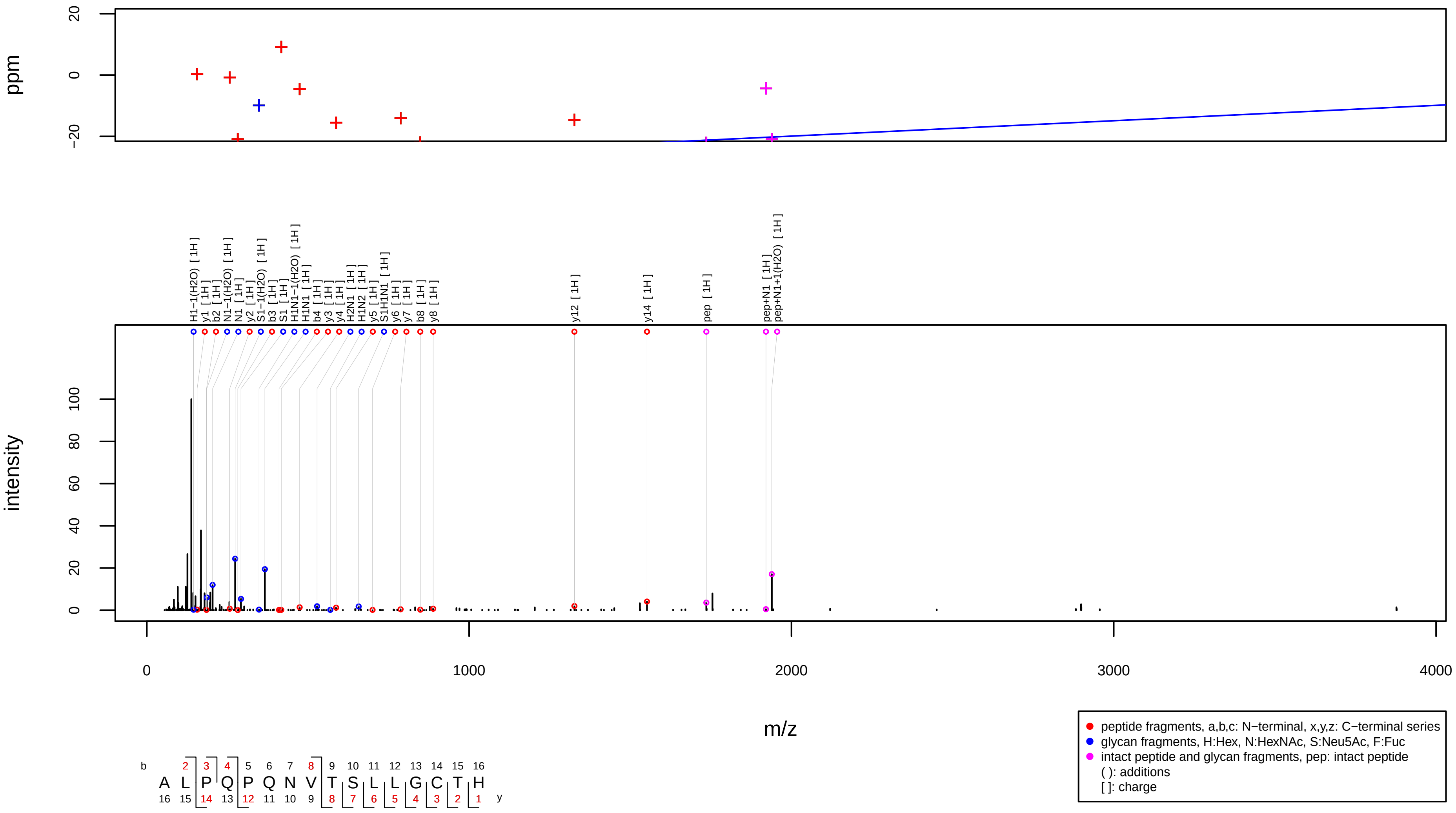

m/z 1203.8142 charge 3 scan 0−0

Score= 77.43 , Hits= 49 , Explained Intensity= 0.54

Peptide: HEMO_HUMAN[181,193]:SWPAVGNCSSALR

Glycan: SHNH(SHNH)HNN, S2H5N4

Charge: 3H

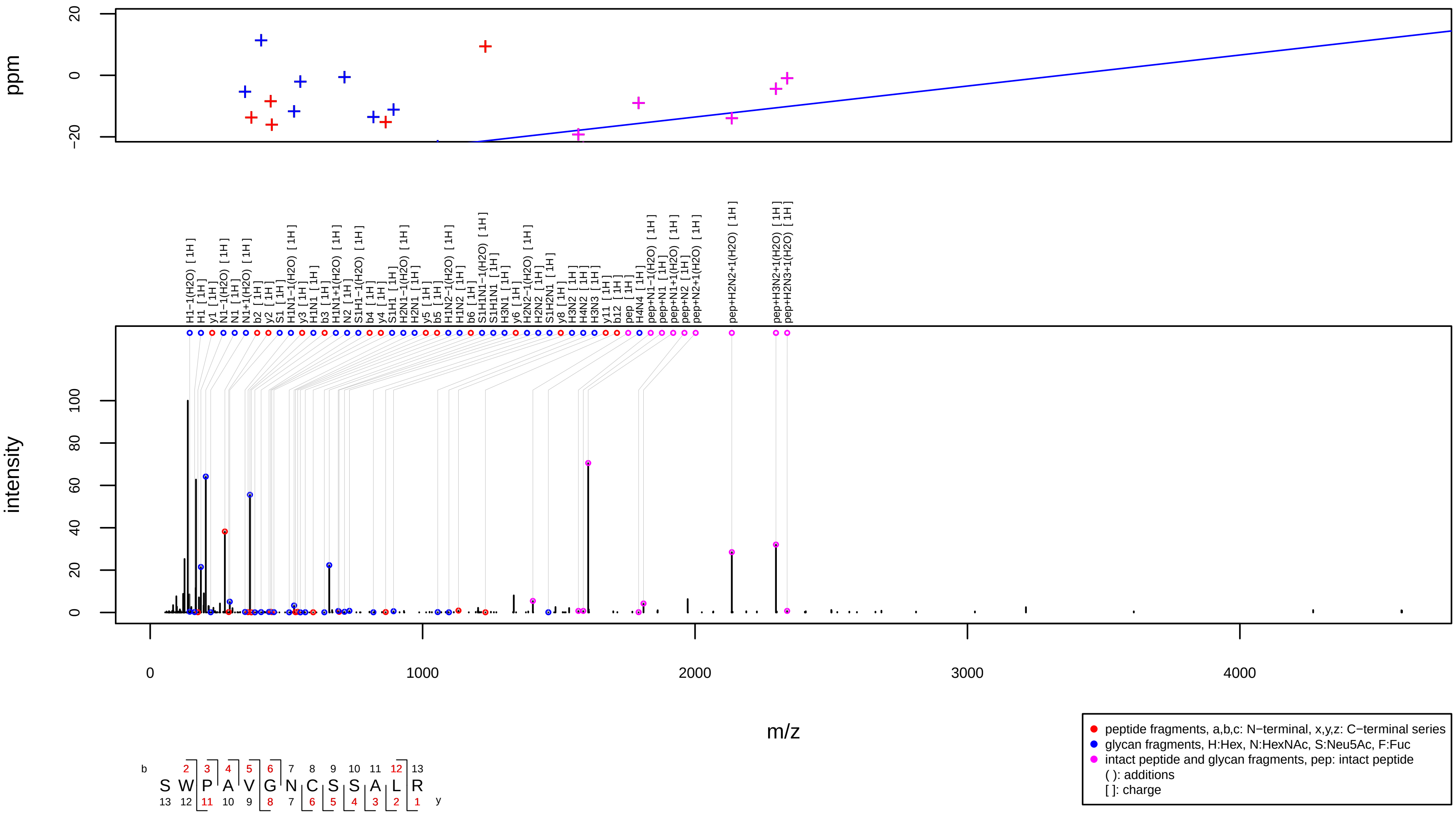

Glycopeptide ID – *Thu Mar 9 13:19:29 2017*

m/z 932.0305 charge 5 scan 0−0

Score= 39.03 , Hits= 33 , Explained Intensity= 0.16

Peptide: HPT_HUMAN[236,251]:VVLHPNYSQVDIGLIK

Glycan: SHNH(SHN(SHN)H)HNN, S3H6N5

Charge: 5H

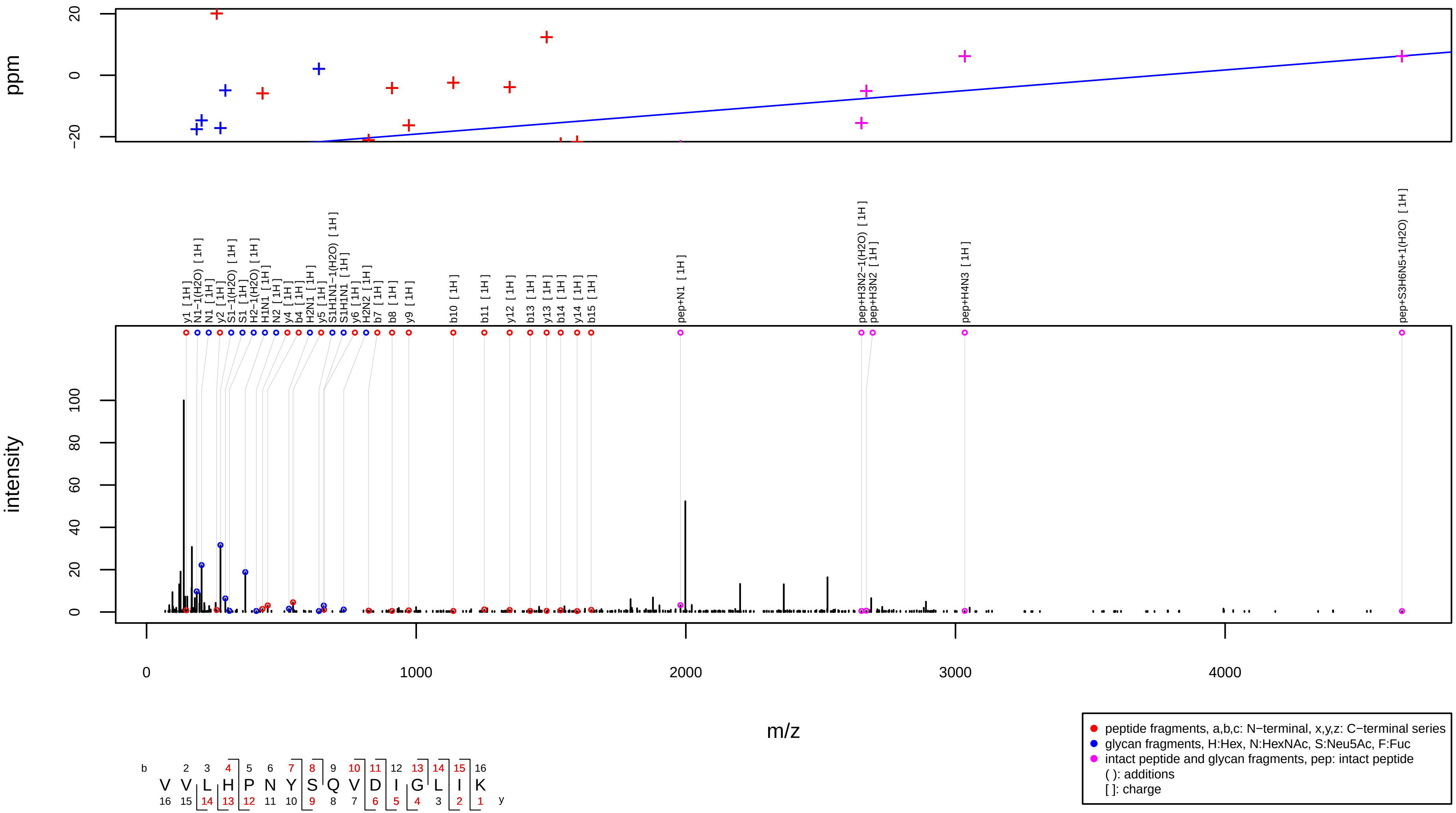

Glycopeptide ID − *Thu Mar 9 13:19:31 2017*

m/z 1092.0148 charge 4 scan 0−0

Score= 39.04 , Hits= 35 , Explained Intensity= 0.16

Peptide: HPT_HUMAN[236,251]:VVLHPNYSQVDIGLIK

Glycan: SHNH(SHN(HN)H)HNN, S2H6N5

Charge: 4H

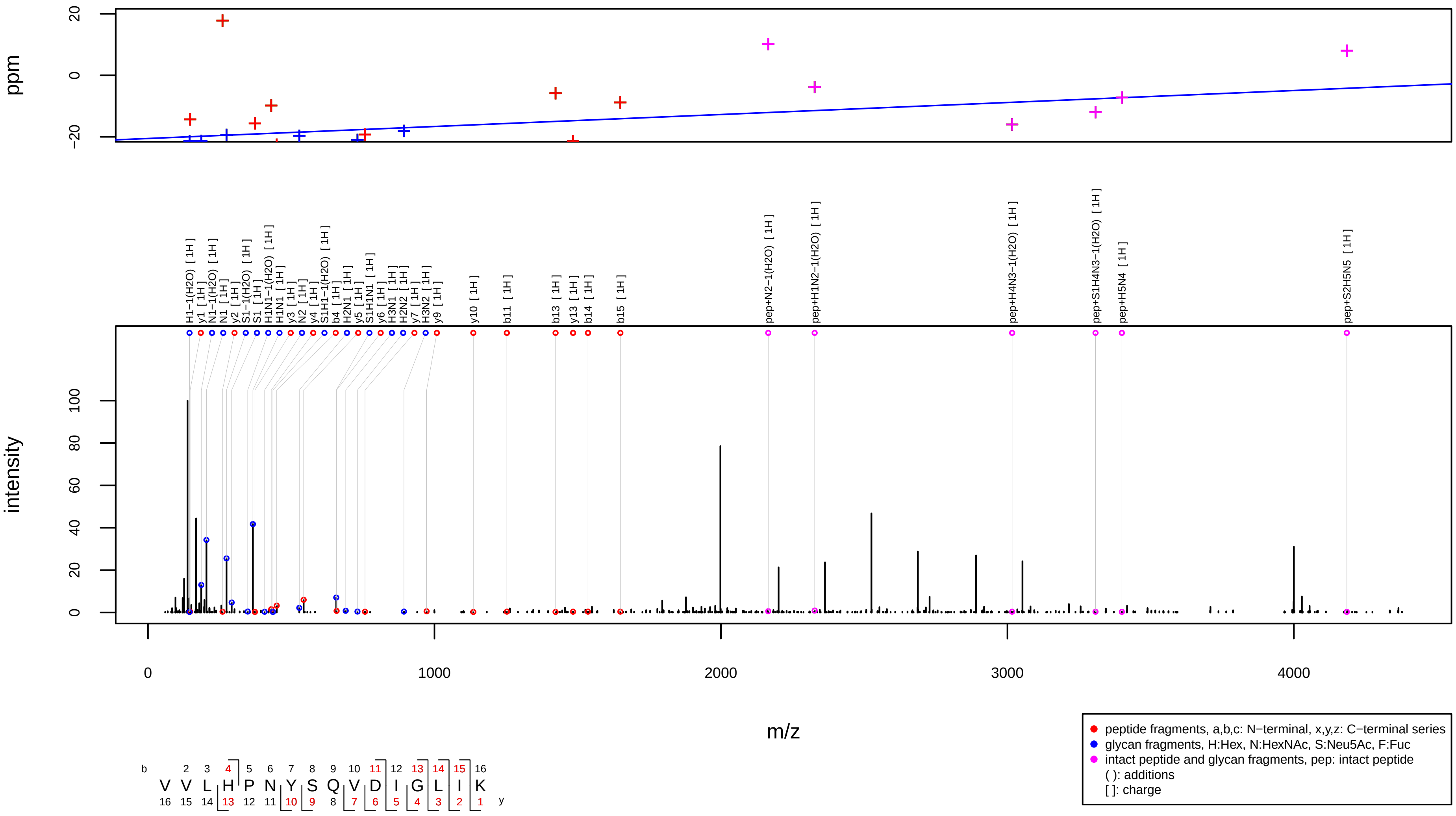

m/z 1115.2258 charge 3 scan 0−0

Score= 58.34 , Hits= 41 , Explained Intensity= 0.18

Peptide: HPT_HUMAN[236,251]:VVLHPNYSQVDIGLIK

Glycan: SHNH(H)HNN, S1H4N3

Charge: 3H

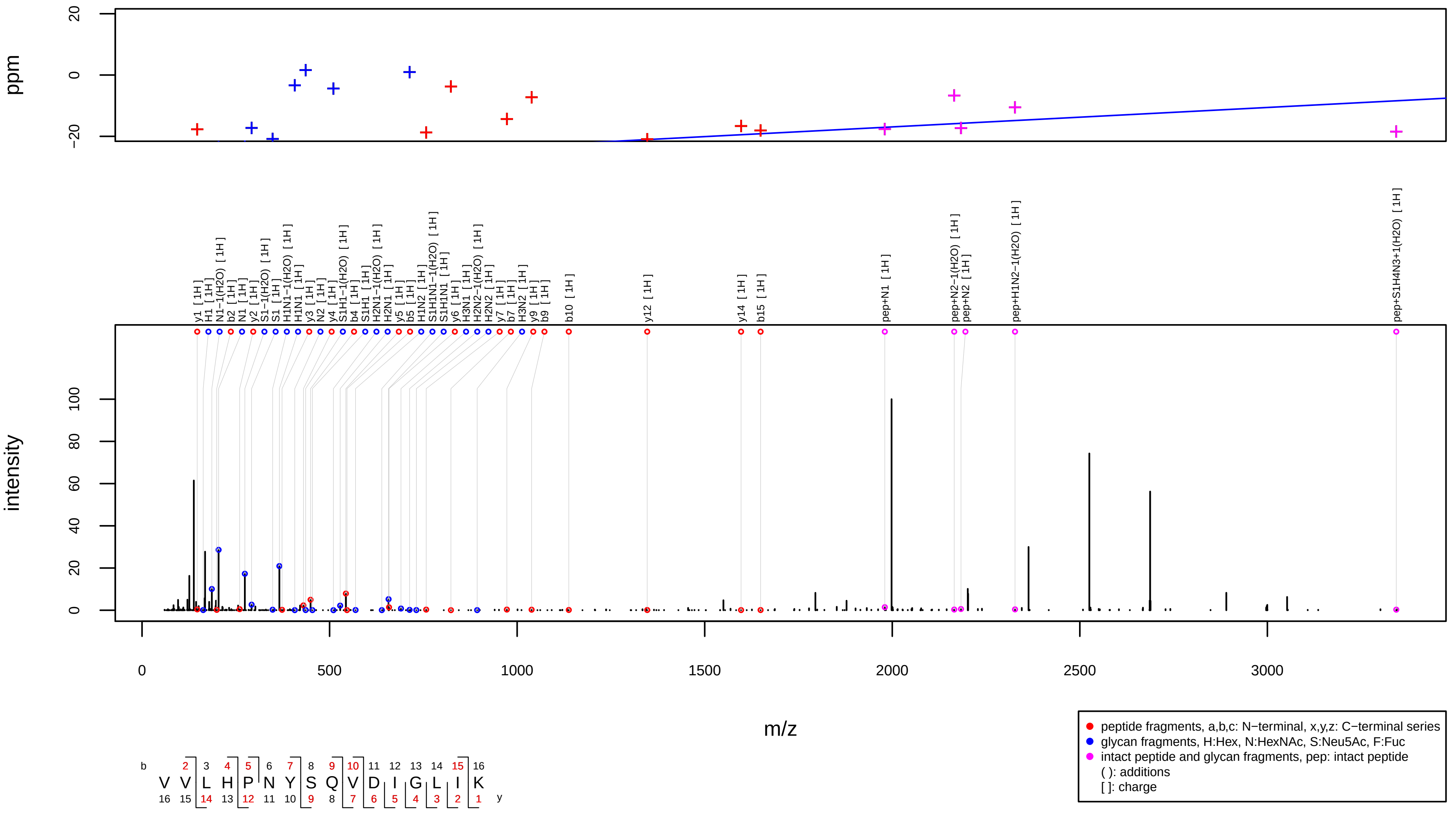

Glycopeptide ID − *Thu Mar 9 13:19:30 2017*

m/z 1225.5516 charge 4 scan 0−0

Score= 42.13 , Hits= 61 , Explained Intensity= 0.15

Peptide: HPT_HUMAN[236,251]:VVLHPNYSQVDIGLIK

Glycan: S2H10N3F2

Charge: 4H

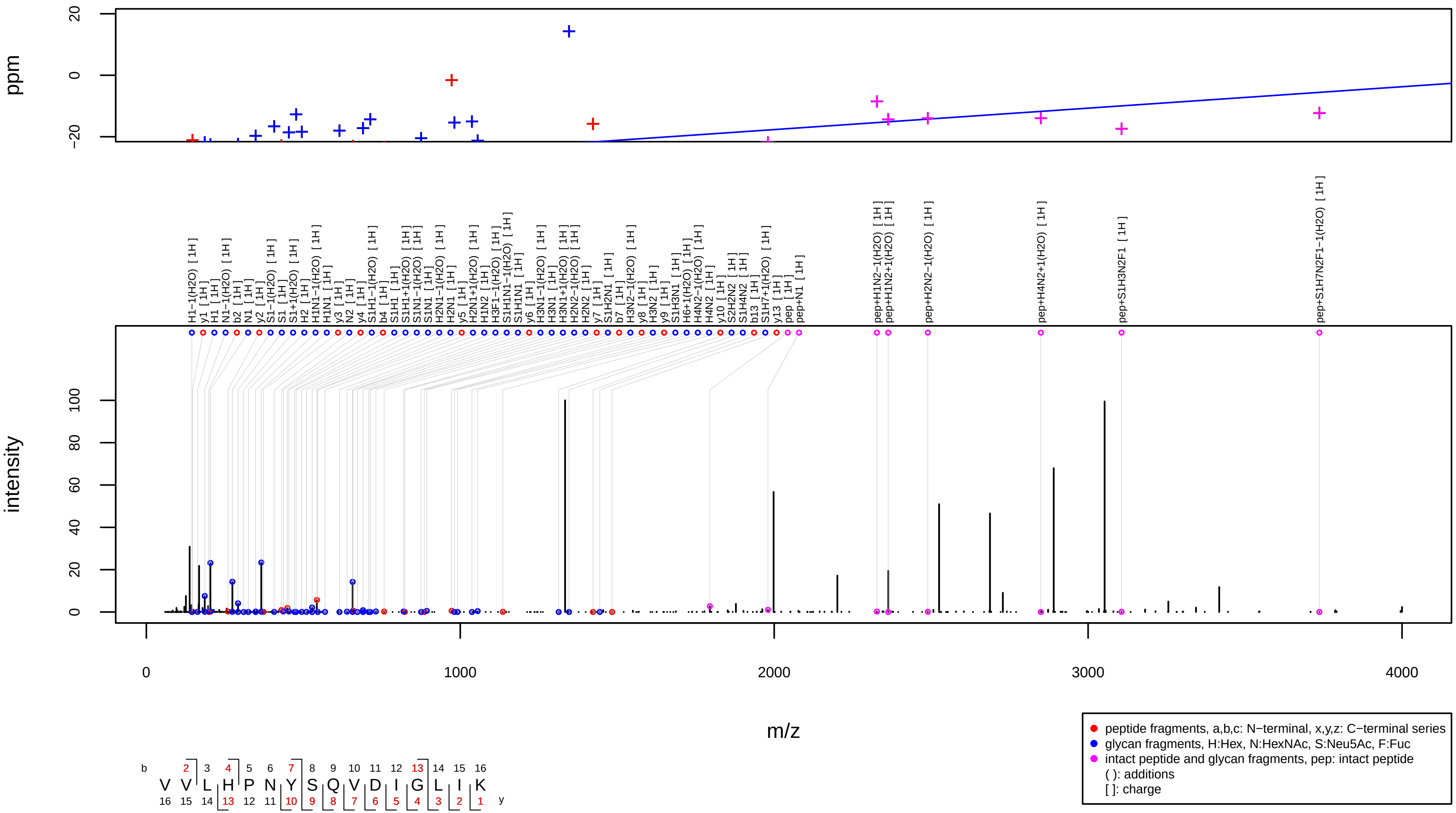

Glycopeptide ID − *Thu Mar 9 13:19:34 2017*

m/z 1333.9701 charge 3 scan 0−0

Score= 74.33 , Hits= 50 , Explained Intensity= 0.17
Peptide: HPT_HUMAN[236,251]:VVLHPNYSQVDIGLIK
Glycan: SHNH(SHNH)HNN, S2H5N4
Charge: 3H

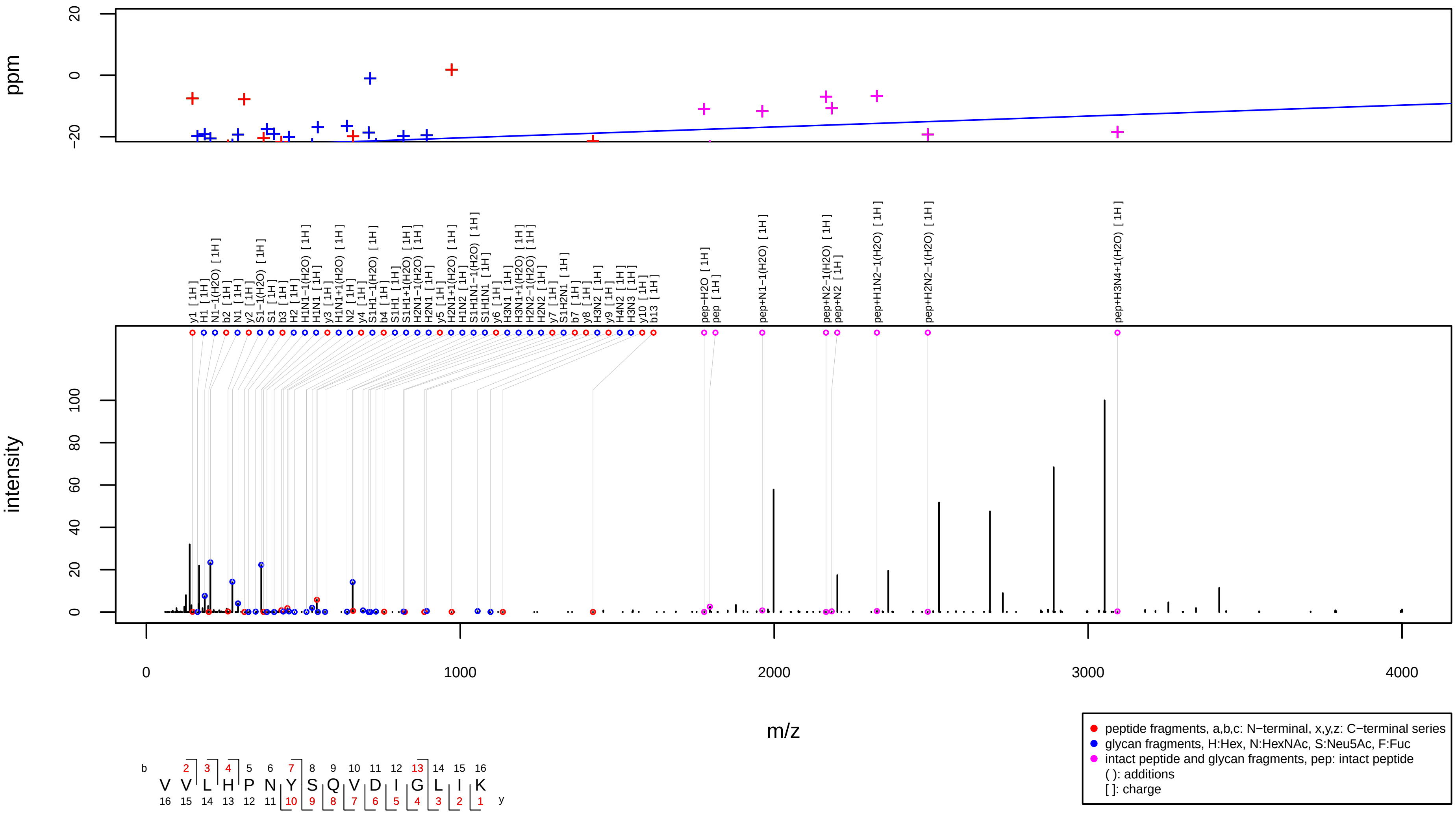

Glycopeptide ID − *Thu Mar 9 13:19:28 2017*

m/z 916.6563 charge 4 scan 0−0

Score= 64.48 , Hits= 44 , Explained Intensity= 0.34

Peptide: HPTR_HUMAN[145,157]:NLFLNHSENATAK

Glycan: SHNH(SHNH)HNN, S2H5N4

Charge: 4H

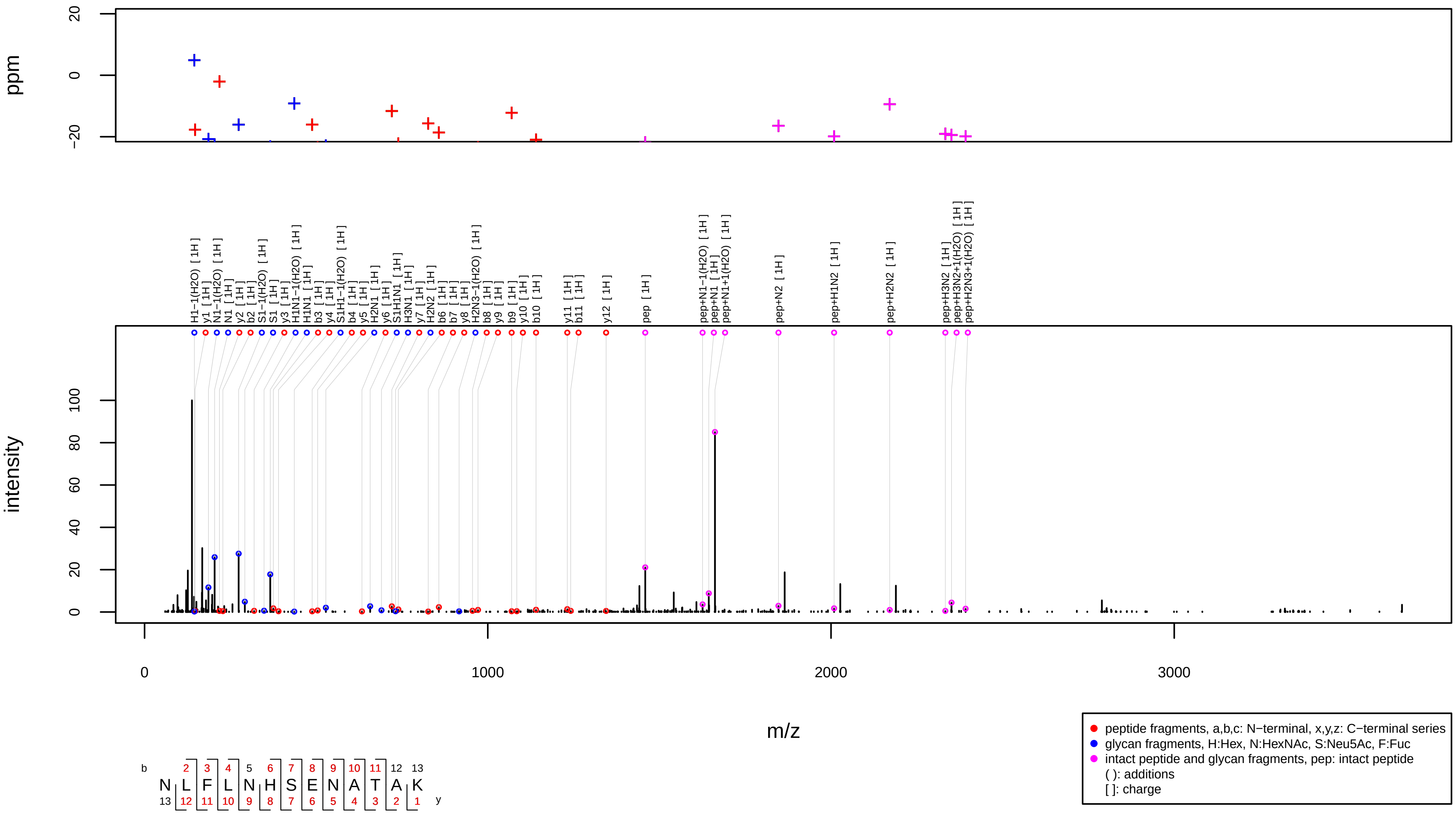

m/z 885.6227 charge 5 scan 0−0

Score= 29.79 , Hits= 23 , Explained Intensity= 0.14

Peptide: IGHA1_HUMAN[127,153]:LSLHRPALEDLLLGSEANLTCTLTGLR
Glycan: HNH(NH)HNN, H4N4
Charge: 5H

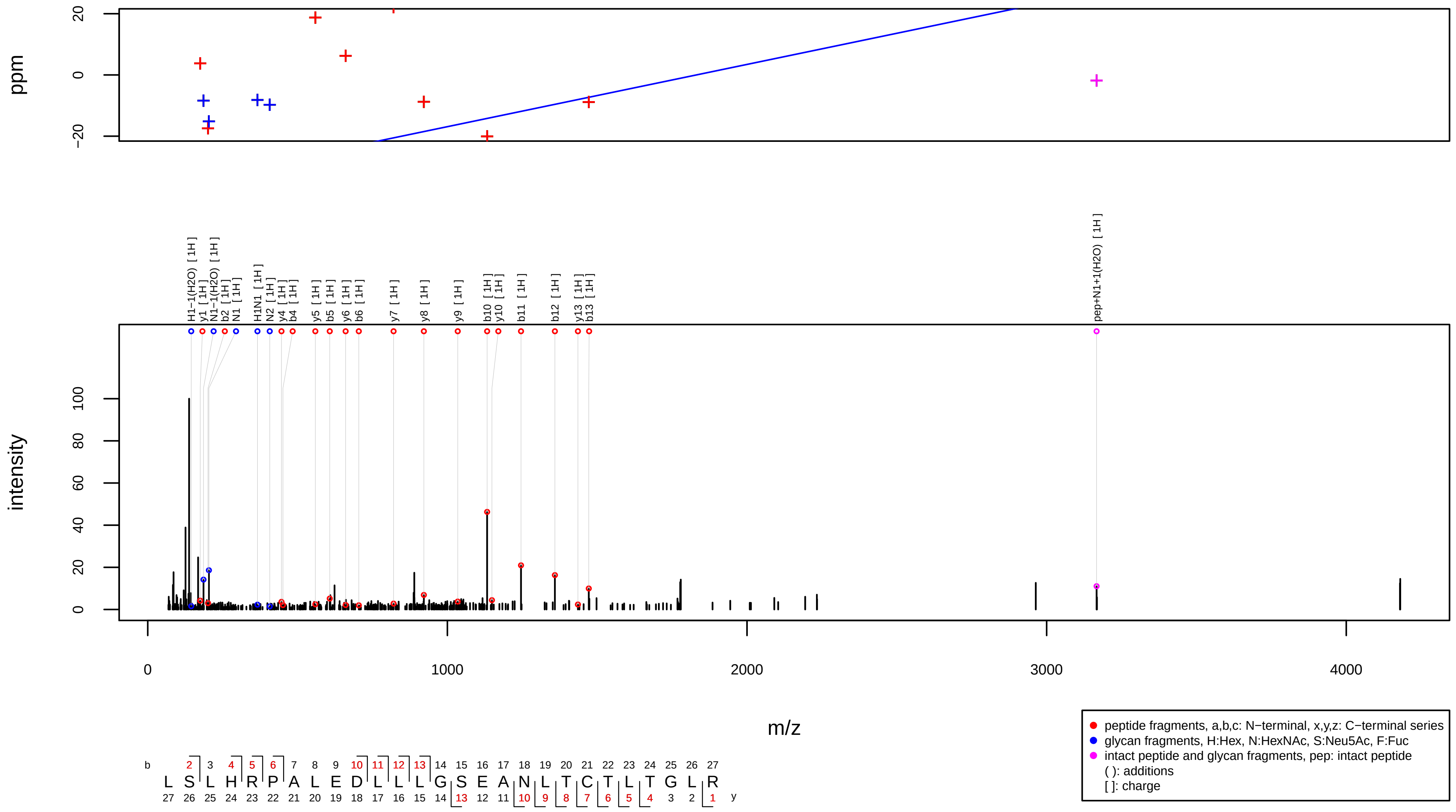

m/z 976.2765 charge 5 scan 0−0

Score= 36.56 , Hits= 31 , Explained Intensity= 0.2

Peptide: IGHA1_HUMAN[127,153]:LSLHRPALEDLLLGSEANLTCTLTGLR
Glycan: SHNH(HNH)HNN, S1H5N4
Charge: 5H

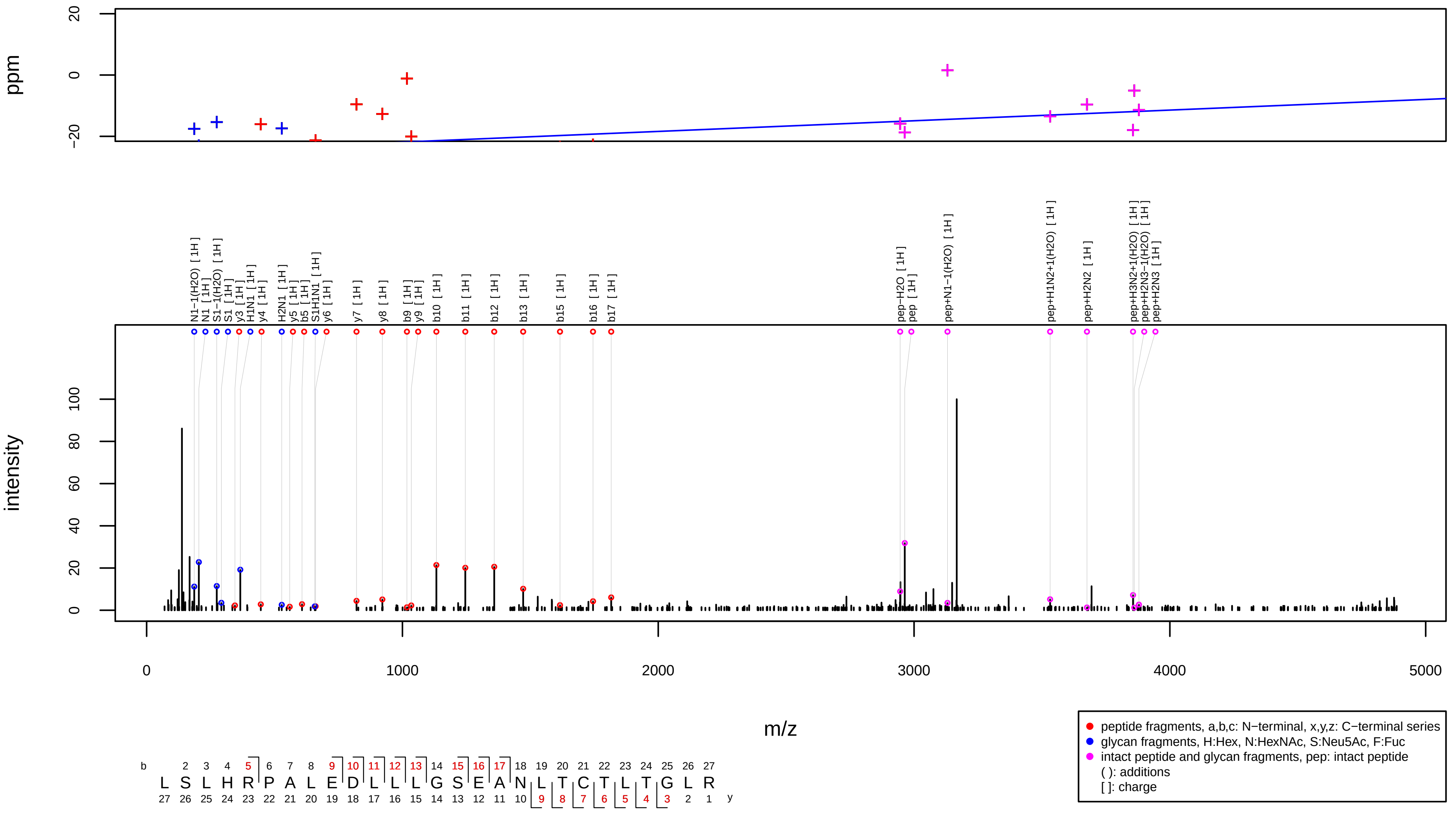

Glycopeptide ID − *Thu Mar 9 13:19:36 2017*

m/z 984.488 charge 5 scan 0−0

Score= 54.39 , Hits= 40 , Explained Intensity= 0.24

Peptide: IGHA1_HUMAN[127,153]:LSLHRPALEDLLLGSEANLTCTLTGLR
Glycan: SHNH(NH)(N)HNN, S1H4N5
Charge: 5H

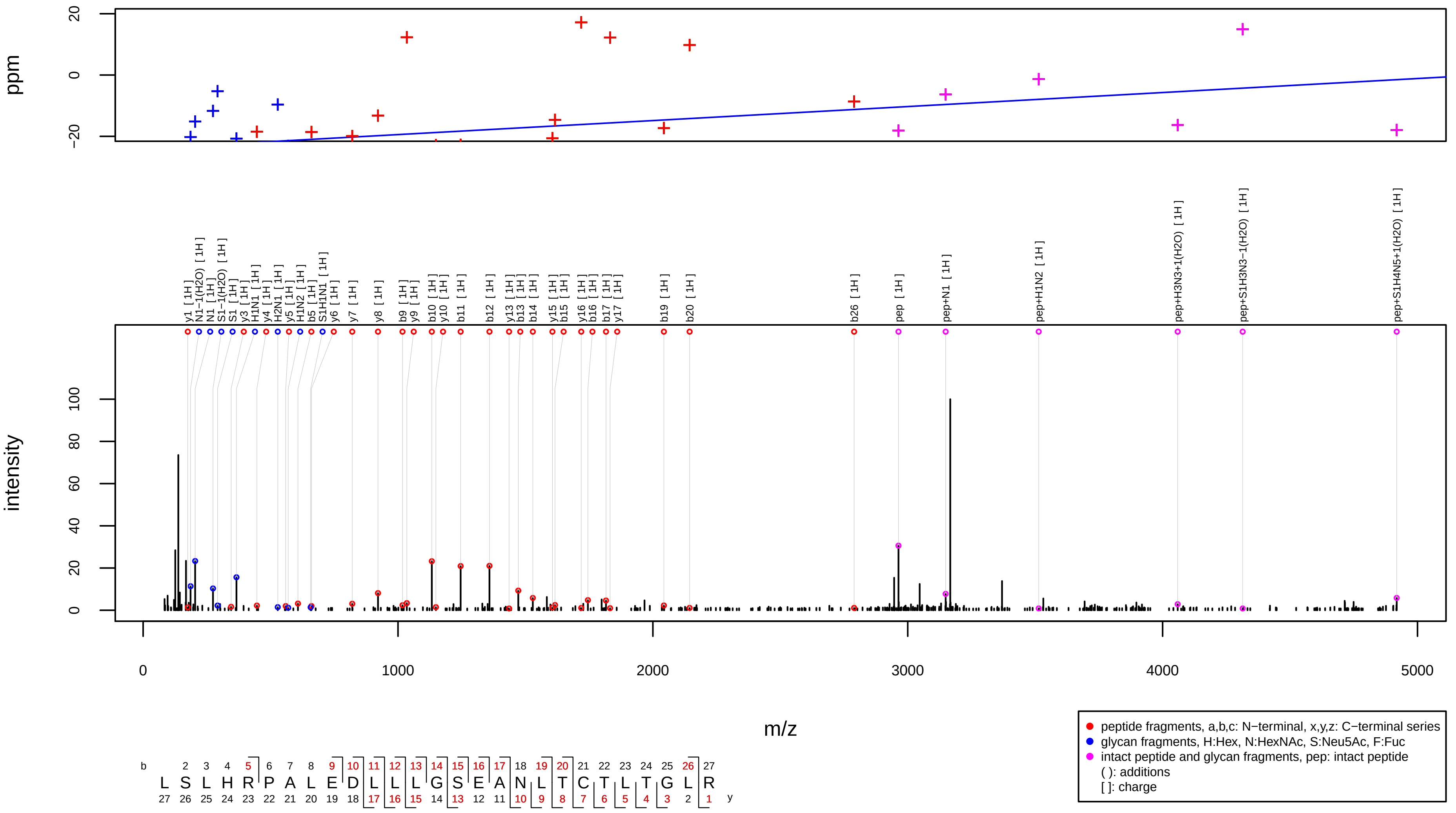

Glycopeptide ID − *Thu Mar 9 13:19:21 2017*

m/z 1016.9038 charge 5 scan 0−0

Score= 64.97 , Hits= 47 , Explained Intensity= 0.26

Peptide: IGHA1_HUMAN[127,153]:LSLHRPALEDLLLGSEANLTCTLTGLR

Glycan: SHN(HN)H(NH)HNN, S1H5N5

Charge: 5H

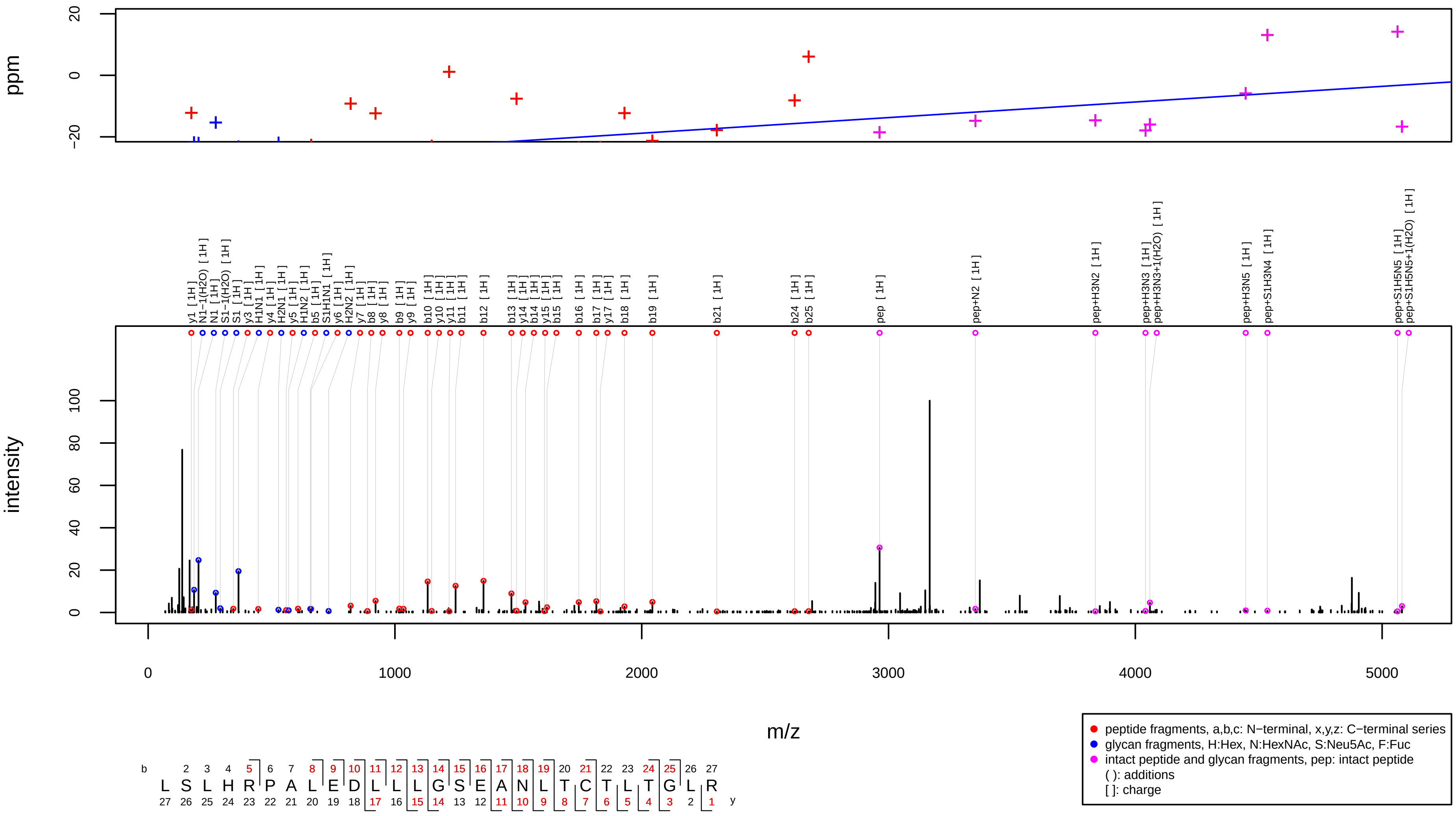

Glycopeptide ID − *Thu Mar 9 13:19:20 2017*

m/z 1147.3264 charge 4 scan 0−0

Score= 33.31 , Hits= 25 , Explained Intensity= 0.18

Peptide: IGHA1_HUMAN[127,153]:LSLHRPALEDLLLGSEANLTCTLTGLR

Glycan: HNH(HNH)HNN, H5N4

Charge: 4H

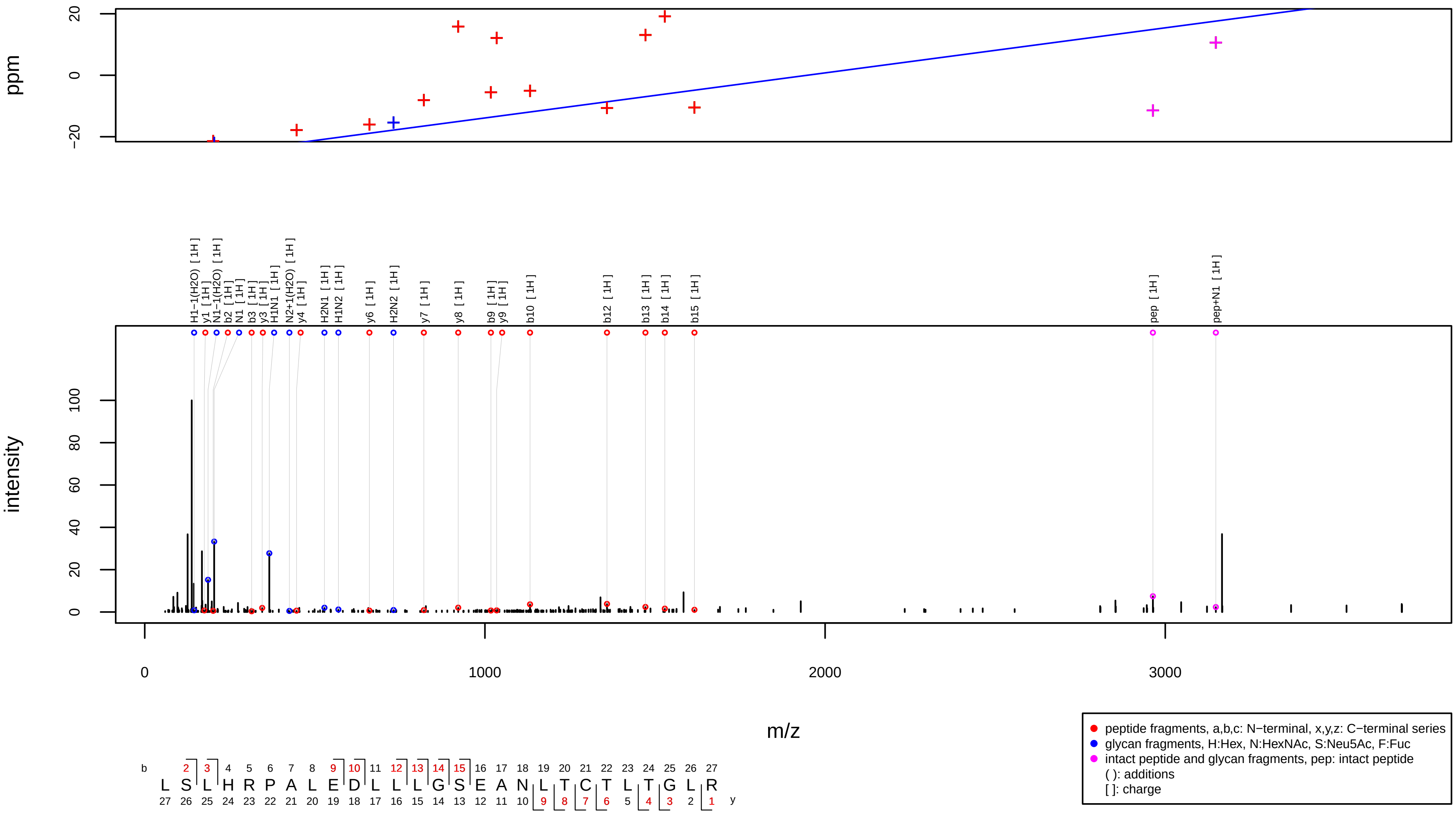

Glycopeptide ID − *Thu Mar 9 13:19:40 2017*

m/z 1179.5922 charge 4 scan 0−0

Score= 52.94 , Hits= 37 , Explained Intensity= 0.22

Peptide: IGHA1_HUMAN[127,153]:LSLHRPALEDLLLGSEANLTCTLTGLR

Glycan: SHNH(NH)HNN, S1H4N4

Charge: 4H

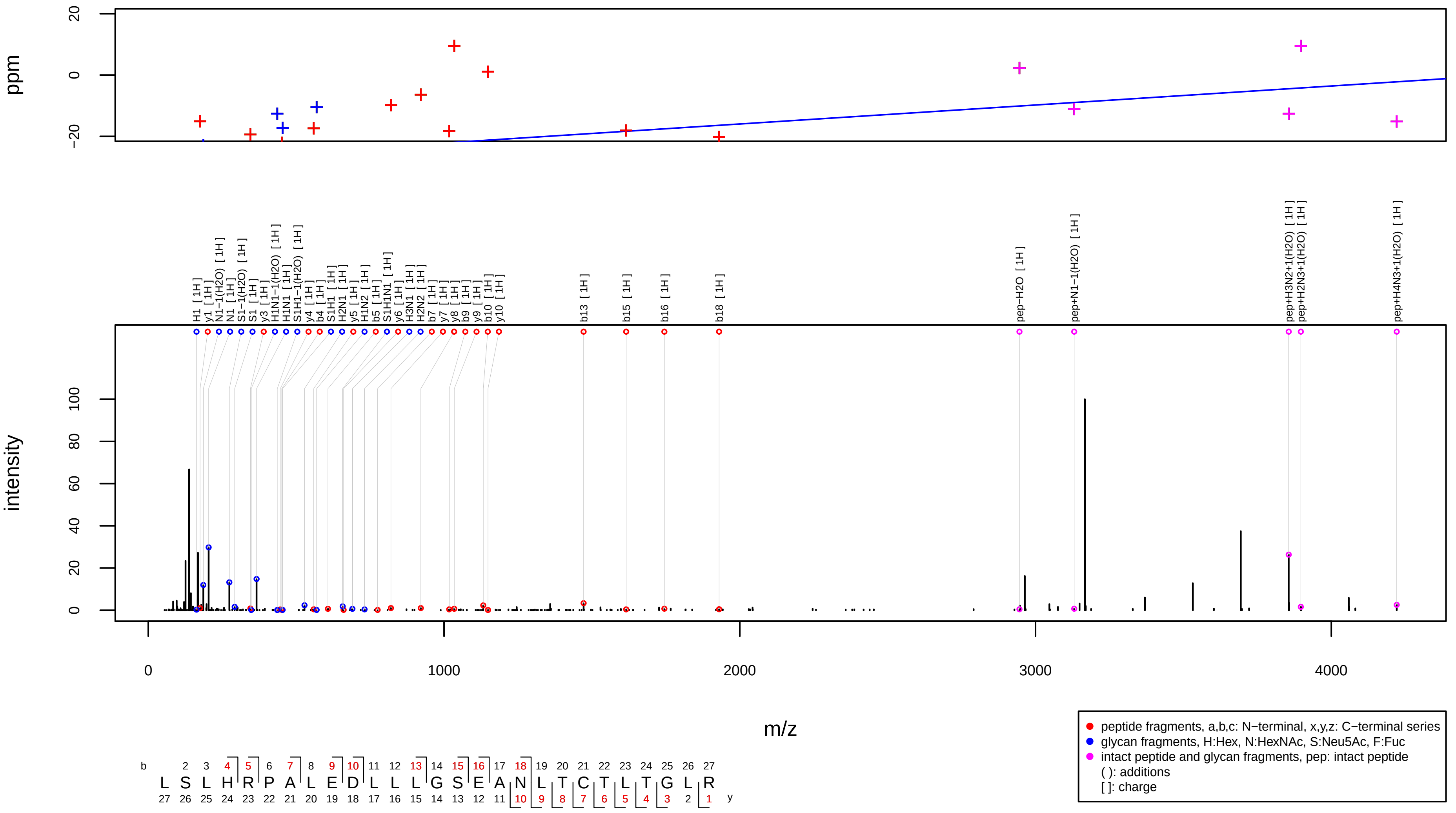

Glycopeptide ID − *Thu Mar 9 13:19:28 2017*

m/z 868.048 charge 3 scan 0−0

Score= 85.11 , Hits= 56 , Explained Intensity= 0.46
Peptide: IGHG2_HUMAN[172,180]:EEQFNSTFR
Glycan: NH(NH)HN(F)N, H3N4F1
Charge: 3H

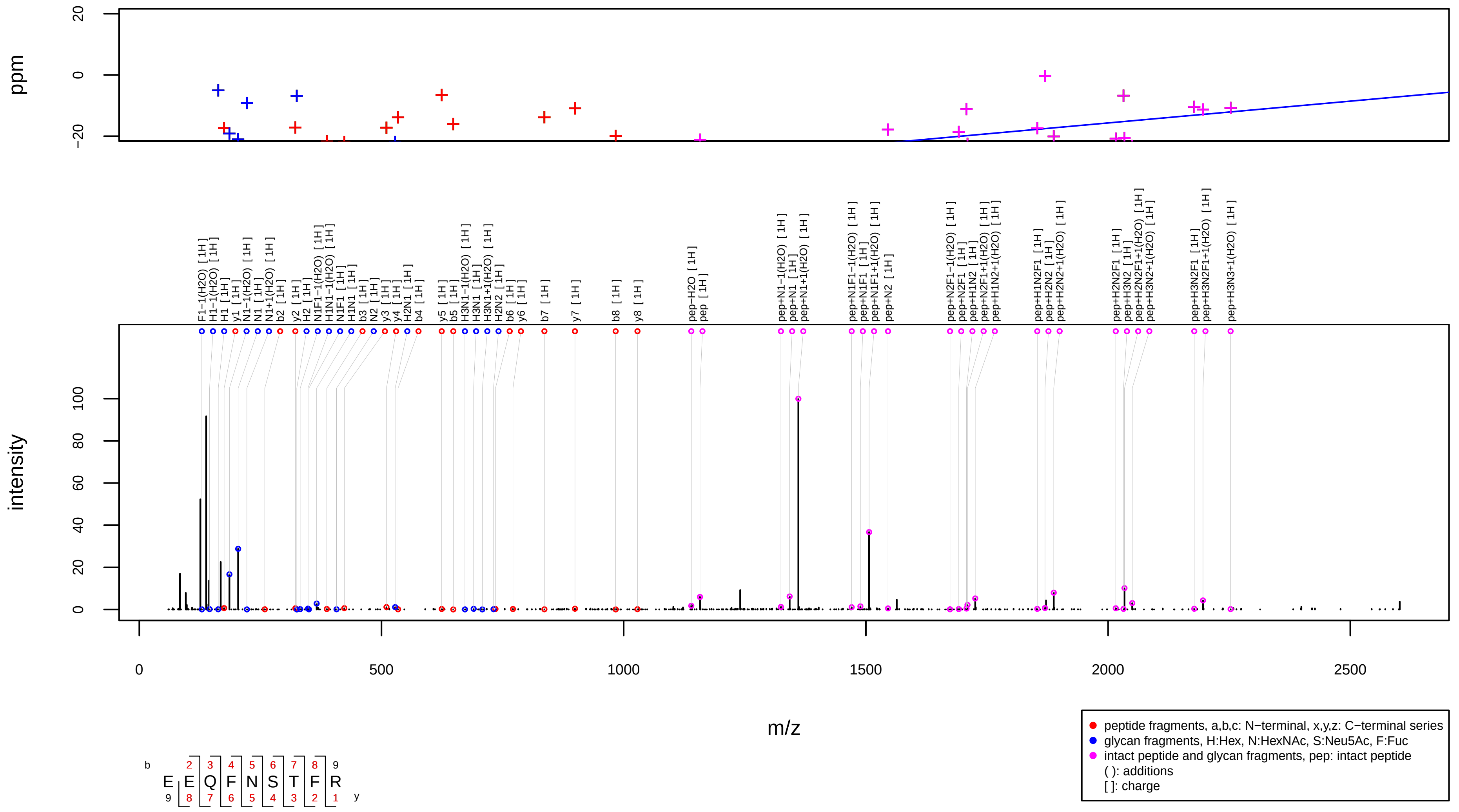

Glycopeptide ID − *Thu Mar 9 13:19:25 2017*

m/z 885.1539 charge 4 scan 0−0

Score= 42.52 , Hits= 37 , Explained Intensity= 0.12

Peptide: IGHG2_HUMAN[168,180]:TKPREEQFNSTFR

Glycan: SHNH(NH)HN(F)N, S1H4N4F1

Charge: 4H

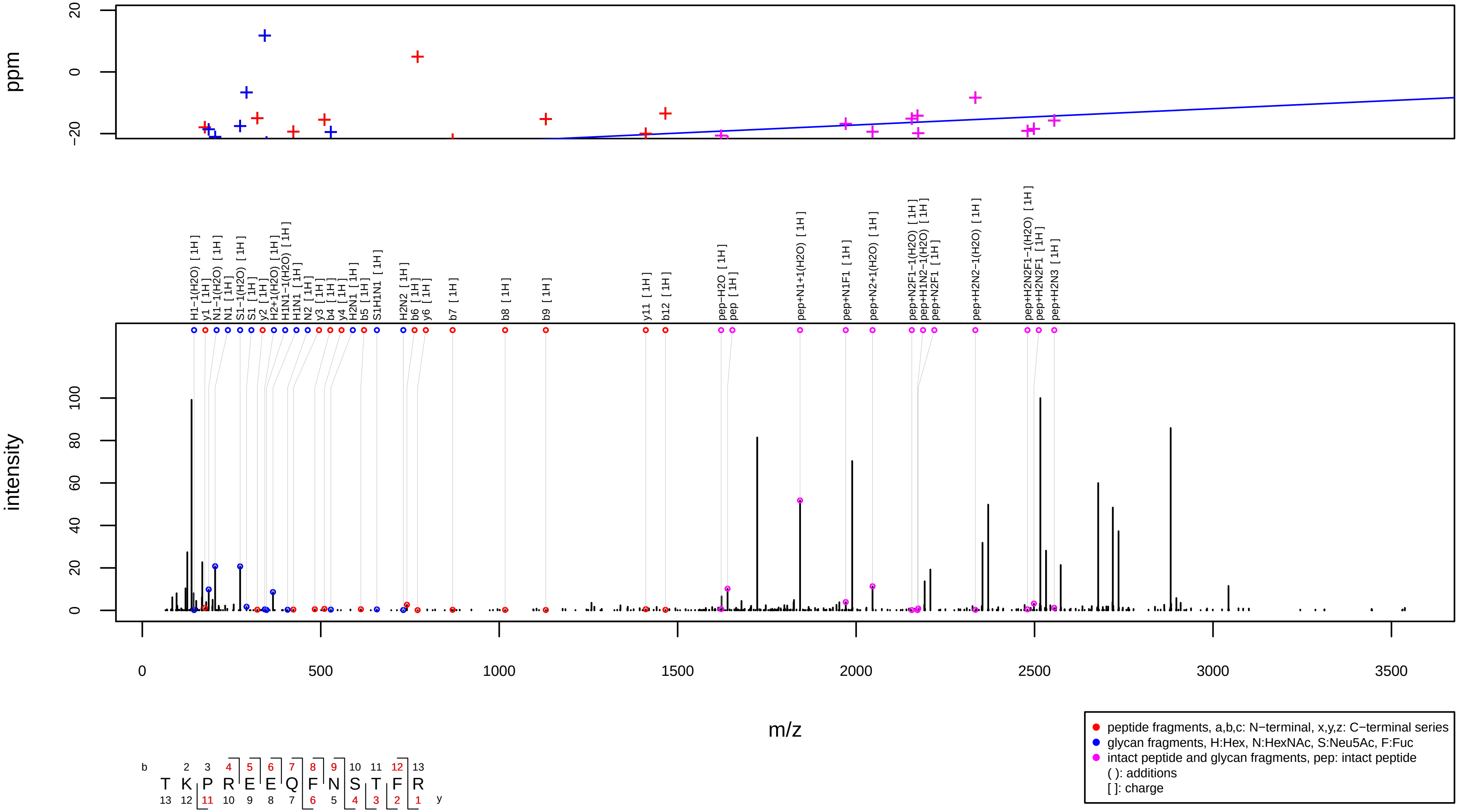

Glycopeptide ID − *Thu Mar 9 13:19:37 2017*

m/z 989.7621 charge 3 scan 0−0

Score= 62.53 , Hits= 48 , Explained Intensity= 0.32

Peptide: IGHG2_HUMAN[172,180]:EEQFNSTFR
Glycan: NNH(HNH)HN(F)N, H4N5F1
Charge: 3H

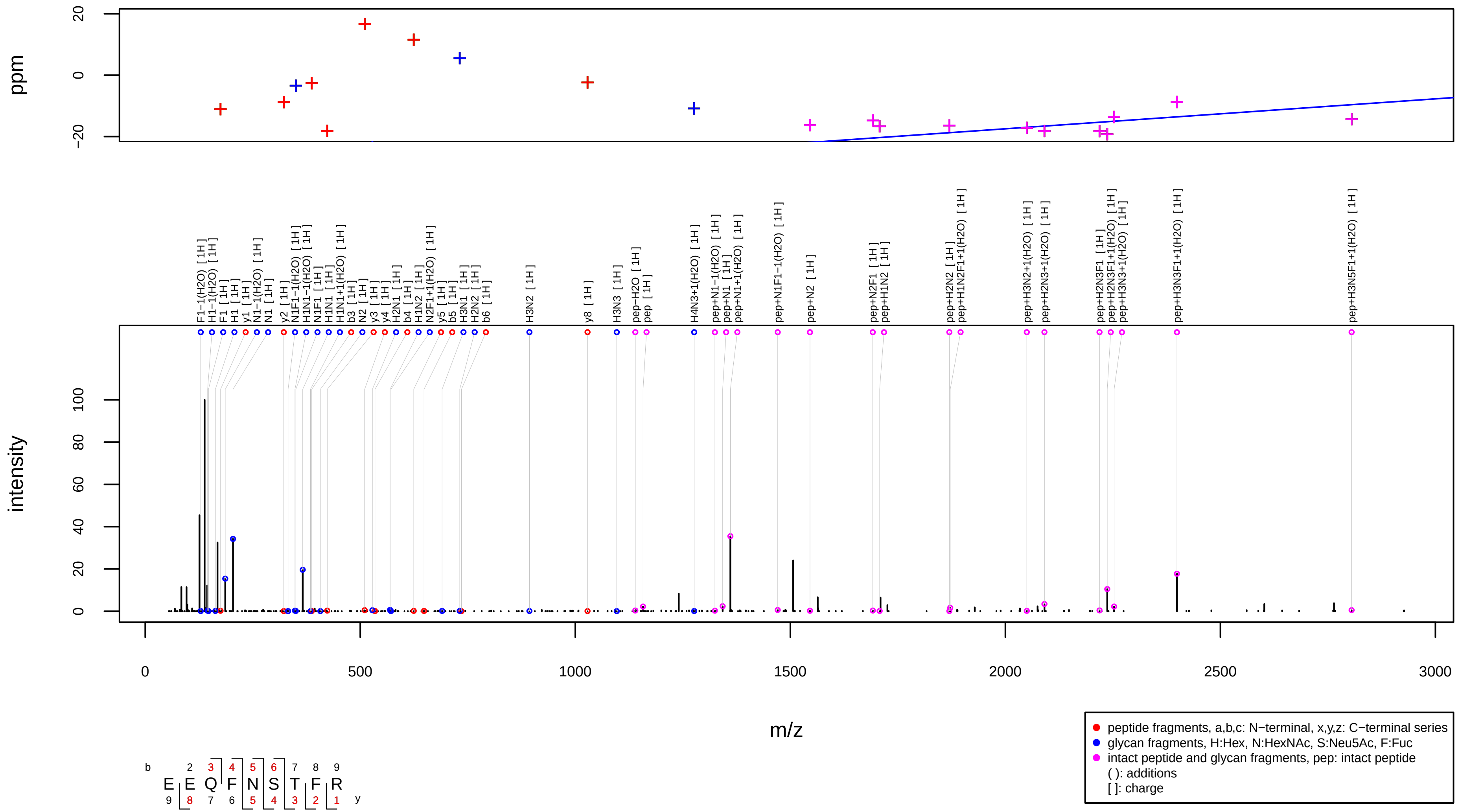

Glycopeptide ID – *Thu Mar 9 13:19:42 2017*
